# A dynamic compartment model for xylem loading and long-distance transport of iron explains the effect of kanamycin on metal uptake in *Arabidopsis*

**DOI:** 10.3389/fpls.2023.1147598

**Published:** 2023-04-18

**Authors:** Ayalew Mentewab, Bethany W. Mwaura, Carla M. Kumbale, Catherine Rono, Natalia Torres-Patarroyo, Tomáš Vlčko, Ludmila Ohnoutková, Eberhard O. Voit

**Affiliations:** ^1^ Biology Department, Spelman College, Atlanta, GA, United States; ^2^ Department of Biomedical Engineering, Georgia Institute of Technology and Emory University Medical School, Atlanta, GA, United States; ^3^ Laboratory of Growth Regulators, Palacký University & Institute of Experimental Botany, Czech Academy of Sciences, Olomouc, Czechia

**Keywords:** kanamycin, iron, zinc, nicotianamine, citrate, WBC19, FRD3, IREG1

## Abstract

*Arabidopsis* plants exposed to the antibiotic kanamycin (Kan) display altered metal homeostasis. Further, mutation of the WBC19 gene leads to increased sensitivity to kanamycin and changes in iron (Fe) and zinc (Zn) uptake. Here we propose a model that explain this surprising relationship between metal uptake and exposure to Kan. We first use knowledge about the metal uptake phenomenon to devise a transport and interaction diagram on which we base the construction of a dynamic compartment model. The model has three pathways for loading Fe and its chelators into the xylem. One pathway, involving an unknown transporter, loads Fe as a chelate with citrate (Ci) into the xylem. This transport step can be significantly inhibited by Kan. In parallel, FRD3 transports Ci into the xylem where it can chelate with free Fe. A third critical pathway involves WBC19, which transports metal-nicotianamine (NA), mainly as Fe-NA chelate, and possibly NA itself. To permit quantitative exploration and analysis, we use experimental time series data to parameterize this explanatory and predictive model. Its numerical analysis allows us to predict responses by a double mutant and explain the observed differences between data from wildtype, mutants and Kan inhibition experiments. Importantly, the model provides novel insights into metal homeostasis by permitting the reverse-engineering of mechanistic strategies with which the plant counteracts the effects of mutations and of the inhibition of iron transport by kanamycin.

## 1 Introduction

Plants mine the soil to extract minerals and in so doing may take up antibiotics, such as kanamycin (Kan), that adversely affect normal plant function. Kan is an aminoglycoside antibiotic produced by the soil bacterium *Streptomyces kanamyceticus*. Aminoglycosides primarily act by binding to the small subunit of prokaryotic and eukaryotic ribosomes, which secondarily leads to inhibition of protein synthesis (Mingeot-Leclercq et al., 1999). Most plants, including *Arabidopsis*, are so sensitive to Kan that this antibiotic is routinely used for the selection of transgenic plants that contain the neomycin phosphotransferase (NPTII) marker. Contrasting this well-documented sensitivity is the discovery that WBC19 (ABCG19) confers Kan resistance (Mentewab and Stewart, 2005). WBC19 encodes for a half-size ATP Binding Cassette transporter of the ABCG family, most members of which are known to function as exporters (Verrier et al., 2008). Knock-out *Arabidopsis wbc19* mutants exhibit strong susceptibility to Kan, while transgenic tobacco plants overexpressing WBC19 gain increased resistance (Mentewab and Stewart, 2005). To date, the underlying mechanisms of resistance are unclear, but appear to be complex. For instance, expression of WBC19 in *Escherichia coli* did not result in a significant increase in Kan resistance (Burris et al., 2008). Furthermore, high levels of expression were detected in cotyledonary leaves in transgenic *Arabidopsis* expressing a *wbc19 promoter*-GUS fusion, as well as in vascular parenchyma of roots, older leaves and flowers (Mentewab et al., 2014). These expression patterns suggest that WBC19 plays a significant role in the long-distance transport of nutrients.

To understand the effects of Kan and the contributions of WBC19, the transcriptome of *Arabidopsis* plants was analyzed upon exposure to Kan (Mentewab et al., 2014). The results indicated an effect of Kan on metal homeostasis, and follow-up metal analysis confirmed significant effects of Kan on metal uptake. Specifically, when control plants were exposed to Kan, Fe uptake was reduced by 60% while Cu levels increased by 50%. This result suggests that a response or consequence of plant exposure to Kan is to limit Fe uptake. Moreover, a comparison of metal levels of control and *wbc19* mutant plants grown in the absence of Kan reveals significant differences in Zn and Cu levels. Specifically, mutant plants had about 50% less Zn and Cu than control plants while Fe and Mn levels remain unaffected. These results suggest that WBC19 plays a role in metal uptake even in the absence of Kan. Lastly, in *wbc19* mutant plants exposed to Kan, Fe levels are even lower than in control seedlings; Cu and Zn levels reach their lowest points, and Mn levels reach significantly lower concentrations. Thus, *wbc19* mutants exhibit the same response as control plants in reducing their Fe uptake and their overall lower metal content is strongly exacerbated by the exposure to Kan. Taken together, these results expose a link between antibiotics and metal homeostasis in *Arabidopsis*.

Fe and Zn are among the most abundant micronutrients found in plants, and their uptake and movement are highly regulated. The major pathway for Fe entry into root epidermal cells in *Arabidopsis* is based on the FRO2/IRT1 system, whereby the ferric chelate reductase 2 (FRO2) reduces Fe(III) chelates to soluble Fe(II) and the iron-regulated transporter 1 (IRT1) subsequently allows its passage into the cells. After entering root epidermal cells, Fe moves *via* plasmodesmata toward the vasculature and is thought to be loaded into the xylem by Iron Regulated1/Ferroportin 1 (IREG1/FPN1) (Morrissey et al., 2009). However, in *ireg1* mutants, Fe transport and signaling are not significantly affected, which leads to the suggestion that redundant transporter(s) exist. Once in the xylem vessels, Fe is chelated by citrate to form a tri-Fe(III) tri-citrate (Fe-Ci) complex that facilitates its long-distance transport (Larbi et al., 2010; Rellán-Alvarez et al., 2010). Critical to this process is the efflux of Ci from the root symplast into the xylem, which is enabled by ferric reductase deficient 3 (FRD3), a Multidrug and Toxin Efflux family (MATE) transporter. FRD3 is expressed in the root pericycle (Rogers and Guerinot, 2002; Green and Rogers, 2004), transports Ci from xylem parenchyma cells into xylem vessels and thereby enables the long-distance transport of Fe throughout the plant (Durrett et al., 2007).

Zn is also taken up by IRT1 and other ZIP family transporters (Grotz et al., 1998; Vert et al., 2001; Connolly et al., 2002). Once in the root symplast, it can either be loaded into the xylem or extruded from root epidermal cells. Loading into the xylem is achieved mainly by two P-type ATPases, namely, heavy metal ATPase 2 and 4 (HMA2 and HMA4) (Hussain et al., 2004; Verret et al., 2004; Eren and Argüello, 2004). Plant cadmium resistance 2 (PCR2) mediates both Zn loading in the xylem and extrusion from root epidermal cells under conditions of excess Zn (Song et al., 2010). Zn can also be sequestered in root vacuoles *via* two transporters of the cation diffusion facilitator family, the metal tolerance protein 1 and 3 (MTP1 and MTP3) (Kobae et al., 2004; Desbrosses-Fonrouge et al., 2005; Arrivault et al., 2006). The presence, transport and compartmentalization of zinc chelators, especially nicotianamine (NA), also play an important role in the uptake and distribution of Zn (Sinclair and Krämer, 2012; Klatte et al., 2009; Haydon et al., 2012; Clemens et al., 2013).

Both Ci and NA have the potential to chelate Fe, Zn and other metals, with the stability of complexes being dependent on pH and stoichiometries (von Wiren et al., 1999). Hence, at the slightly acidic pH of the xylem, Ci is the main chelator of Fe, while NA is thought to be the main chelator of Fe at a neutral pH in the cytoplasm. NA is also considered the main chelator of Zn and Cu in the xylem.

Many components of metal homeostasis, and especially of Fe and Zn uptake, have been characterized. Yet gaps remain regarding the identity of the potentially redundant iron transporter(s) and how NA is loaded in the xylem. Moreover, the role of WBC19 and the effect of Kan are yet to be integrated into our understanding of metal uptake. To this end, we propose a conceptual model according to which iron loading in the xylem is dependent on an Fe-Ci transporter that is yet to be identified, as well as on WBC19, which appears to transport Fe-NA and possibly other metal-NA complexes, including Zn-NA or NA itself. Importantly, this model stipulates that the Fe-Ci transporter is inhibited in the presence of Kan, at which point WBC19 seems to serve as the major Fe transporter. To permit quantitative analysis of this proposal, we convert the conceptual model into a dynamic compartment model (see **Supplementary material**). First, we use general and specific knowledge about metal uptake in *Arabidopsis* to devise an interaction diagram on which the construction of the dynamic model is based. This model is formulated as a slightly generalized mass-action system that allows inhibition in the form of power-law functions (Voit, 2013; Voit et al., 2015). This format is arguably the simplest and least biased. In the second step, we use our time series data, described in the experimental sections of this article, to parameterize this model. Third, we perform a computational analysis of the model, which allows us to interpret and explain the observed differences between data from wildtype and from mutation and inhibition experiments in terms of physiological strategies with which the plant responds to these alterations.

## 2 Materials and methods

### 2.1 Experimental methods

Our study used *Arabidopsis* lines in which WBC19, IREG1 and FRD3 were mutated. The *wbc19* and *ireg1* mutants were from the SALK collection (SALK_107731 and SALK_145458) and described in Mentewab and Stewart (2005) and Morrissey et al. (2009) respectively. We furthermore checked that homozygous *ireg1* mutants expressed the NPTII (neomycin phosphotransferase II) protein, using an ELISA Kit from Agdia. Given the expression of NPTII in the *wbc19* and *ireg1* mutants, a line that expresses the NPTII gene but without disruption of genes was used as a control (SALK_064816C), as in Mentewab et al. (2014). The *frd3* mutants in this study are the single-nucleotide EMS mutant *frd3-2* lines described in Rogers and Guerinot (2002). WT Col1 seeds were used for comparison with these *frd3* mutants.

Plants were grown on MS medium with Kan at a concentration of 0, 15 or 50 mg/l. Media at 50mg/l kanamycin were used for the SALK mutants expressing NPTII. For *frd3* mutants and WT col1, which did not have an NPTII gene insertion, the lower threshold of Kan of 15mg/l was used. Twenty-two milliliters of medium were poured in each 11 cm diameter Petri dish.

Seeds were sterilized and 52 seeds were placed on each plate. Plates were kept at 4°C for 3 days before they were moved to a growth chamber at 22°C, with a 16-hour light cycle. Plants were grown for 7, 10 or 13 days before images were captured. Plants were collected and rinsed four times with distilled water. During harvest of 7, 10, 13 days-old plants for metal analysis, material from 6, 4 and 2 plates respectively were pooled for each replicate. A total of 3 replicates were used for each experimental condition. Plant material was dried for 3 days in a 75°C oven, weighted and shipped to the Plant, Soil and Water Analysis Laboratory at the University of Georgia for elemental analysis by ICP-MS.

For root length measurements, 10 seeds were placed in 2 replicate plates placed vertically in the growth chamber. Plate images were captured after 6 and 8 days and root lengths extracted using Image J software.

### 2.2 Design of a computational model of metal uptake and antibiotic resistance in Arabidopsis

**Figure 1:**
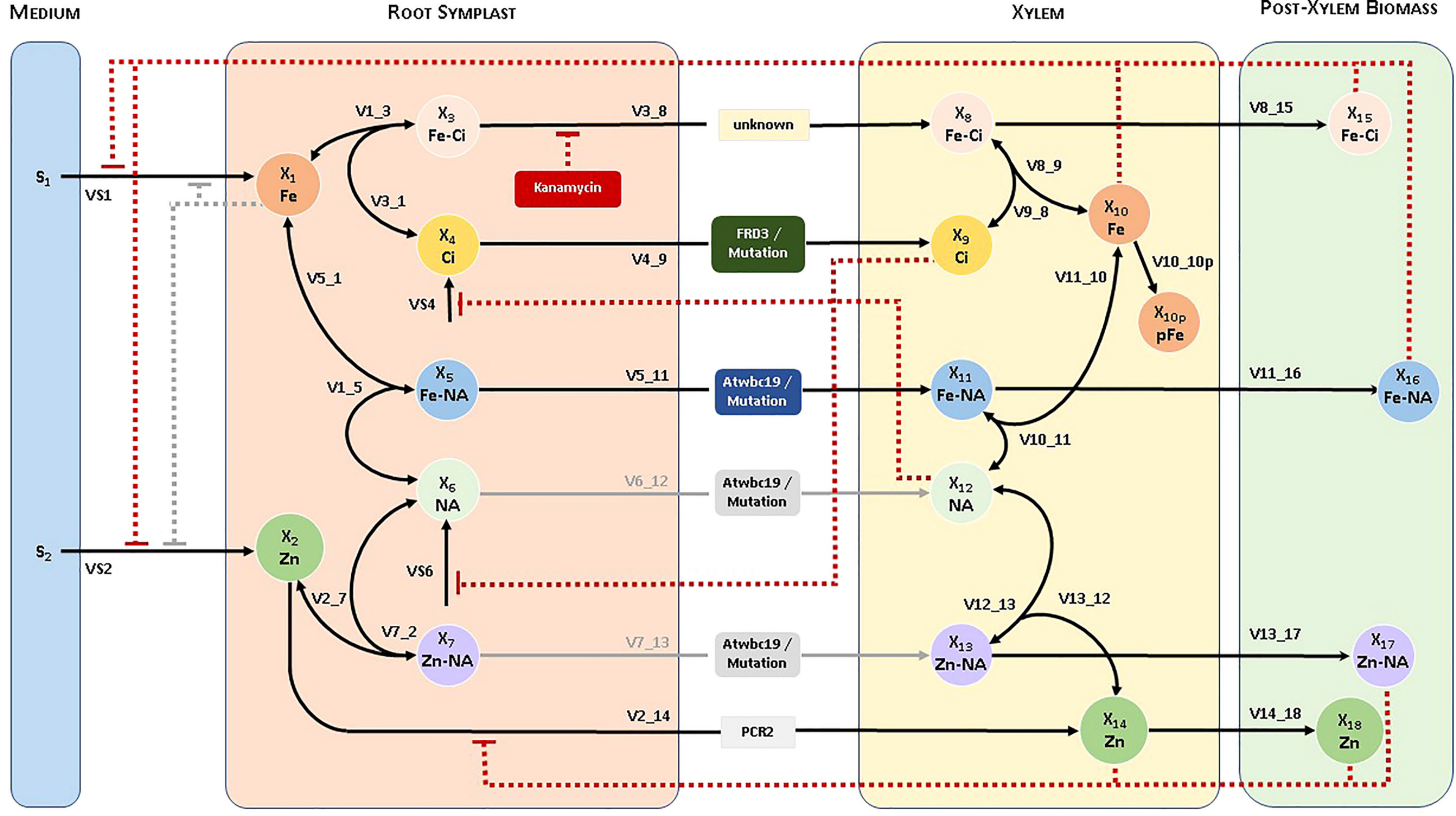
Diagram of the pathway system, and naming of variables for the Fe and Zn uptake system in *Arabidopsis*. The ability of WBC19 to transport Zn-NA or NA is not critical to the model and therefore represented by grey lines. Similarly, initially considered but ultimately unimportant regulatory signals are shown in grey.

Our modeling effort proceeded in three distinct steps; for details, see **Supplementary Material**. In the first, we created a conceptual model of the system, which is represented by a diagram consisting of boxes—one per variable—and arrows—representing material transport among boxes, as well as regulatory signals (**Figure 1**). This initial design process is almost entirely based on biological knowledge of the system, combined with data and other information that are available to formulate and calibrate the model. The resulting diagram is of great importance because it permits the visual checking of potential inconsistencies between the model structure and reality.

The model includes the dependent variables as shown in **Table 1**. In addition, *S*_1_ and *S*_2_ are independent variables denoting Fe and Zn in the medium, respectively, while *S*_4_ and *S*_6_ describe Ci and NA generated in the symplast. Fluxes and transport steps between any two dependent variables with indices *p* and *q* are denoted with V*p*_*q*. As an example, the transport of Fe from root symblast (*X*_3_) into xylem (*X*_8_) is denoted as V3_8 (see **Figure 1**). For uptake fluxes, we use the simplified notation VS1, …, VS4.

**Table 1:** Dependent variables in the model depicted in **Figure 1**.

Root Symplast	Xylem	Post-Xylem Biomass
*X*_1_: Fe		
*X*_2_: Zn		
*X*_3_: Fe-Ci	*X*_8_: Fe-Ci	*X*_15_: Fe-Ci
*X*_4_: Ci	*X*_9_: Ci	
	*X*_10_: Fe *X*_10_ *_p_*: precipitated Fe	
*X*_5_: Fe-NA	*X*_11_: Fe-NA	*X*_16_: Fe-NA
*X*_6_: NA	*X*_12_: NA	
*X*_7_: Zn-NA	*X*_13_: Zn-NA	*X*_17_: Zn-NA
	*X*_14_: Zn	*X*_18_: Zn

In the second step, the conceptual model is converted into a symbolic model, in which functional representations for all processes are chosen. In the third step, this symbolic model is parameterized to yield a complete computational representation of the actual system. This fully parameterized model can be used for simulation analyses and explorations of compensatory mechanisms accompanying the mutants and Kan exposure. Details of the technical steps toward a fully parameterized model are presented in the **Supplementary Material**.

#### 2.2.1 Design of a conceptual model

Our experimental observations and general knowledge regarding metal uptake led us to propose a concise conceptual model, which we visualized in the form of a system diagram (**Figure 1**). We distinguish four compartments, namely the growth medium, root symplast, xylem and remaining post-xylem biomass.

The seedlings take up Fe and Zn (among other compounds that are not of immediate interest here) from the medium into the root symplast and synthesize Ci and NA within. We distinguish six transport processes from the root symplast into the xylem, from where material is subsequently distributed throughout the plant. Each of these processes could consist of several parallel processes in the actual plant. Fe-Ci and Ci are transported into the xylem by means of an unknown transporter and FRD3, respectively. Fe can also form a complex with NA within the root, and this complex is transported into the xylem by WBC19. In parallel, Zn is transported directly to the xylem by PCR2, HMA2, HMA4 and possibly as Zn-NA chelate by WBC19. WBC19 could also transport NA by itself, but the ability of WBC19 to transport Zn-NA or NA is not critical to the model.

The observations leading to this model and an understanding of the link between metal uptake and Kan resistance are as follows. First, given the vascular localization of WBC19, long distance transport of Fe appears to be involved in the mechanism of antibiotic resistance. Second, control plants exposed to Kan significantly decrease their Fe uptake into the xylem, yet do not display the chlorosis or small roots that are typically associated with exposure to Kan in *wbc19* mutants. Thus, it appears that lowering Fe uptake into the xylem is a coping or trade-off mechanism for resistance to Kan. A possible explanation for such a response is that Fe and Kan can move through the same Fe-Ci transporter. This notion is supported by the observation that the Fe-Ci complex appears to have structural similarities with Kan and the ability to move through the same transporter (Conte and Lloyd, 2010). This speculation leads us to propose that the Fe-Ci transporter is inhibited by Kan.

*Wbc19* mutants exhibit a decreased uptake of Zn and Cu, which suggests that WBC19 contributes to the transport of these metals, either directly or in the transport of their main chelator NA or possibly their chelated forms (Mentewab et al., 2014). Given its role in metal transport and Kan resistance, we propose that WBC19 is an alternate route for Fe loading into the xylem, by means of transporting Fe-NA, especially if the primary Fe-Ci transporter is inhibited by Kan. Specifically, if this hypothesis is correct, WBC19 would transport Fe-NA into the xylem. However, Fe-NA in the xylem would be short lived as Ci would quickly remove Fe^2+^ from NA, triggering rapid oxidation of iron into Fe^3+^-Ci complexes (von Wiren et al., 1999). WBC19 could possibly also transport Zn-NA and Cu-NA into the xylem. The model works without this possibility but cannot exclude it.

**Figure 2:**
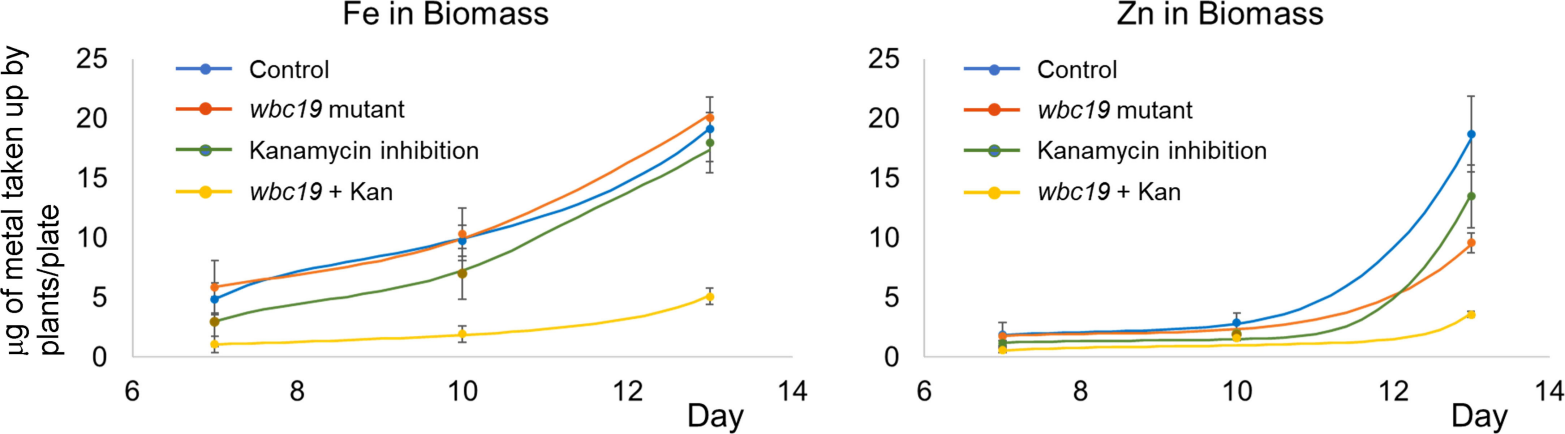
Time courses of total iron and zinc content of control and *wbc19* seedlings grown on MS medium with or without 50mg/l of Kan. The dots and error bars represent the experimental measurements, averaged from three replicates, and their standard deviations. The lines show model results.

The model accounts for regulatory mechanisms known to affect metal homeostasis. Biomass affects Fe uptake by the IRT1 and FRO2 system in two ways. First, the growing plant requires increased metal uptake for exponential growth, as it is clearly reflected in the strongly increasing amounts of Fe and Zn in the biomass (**Figure 2**) during this early phase of the plant’s life. Because the model does not account for biomass per se, this activation is represented in proxy as feedback by the amounts of Fe and Zn in the biomass. At the same time, iron uptake by the IRT1 and FRO2 system is subject to local and long distance signaling (Vert et al., 2003). The model analysis leaves no doubt that the overall effect of biomass on uptake is positive (see **Supplementary Material**) in the young plant; this situation might be different in older plants when exponential growth slows down. We also tested the hypothesis of local negative feedback of metal uptake by the Fe in the roots, but this mechanism caused undue restriction of metal uptake.

We furthermore integrated what is known about the indirect effects of low NA or Ci in the xylem. For instance, if WBC19 is mutated, NA in the form of Fe-NA or possibly Zn-NA or NA cannot be transported into the xylem. Indirectly, this mutation would enhance Ci transport, which reflects the findings of Schuler et al. (2012), who demonstrated a large increase (from 200 to 900 μM) of xylem citrate levels for mutants unable to synthesize NA (*nas4X-2*). Analogously, we made the assumption that NA transport will increase if Ci transport is reduced. This assumption is supported by a reported two-fold increase in NA levels when Ci loading is inhibited, as is the case for *frd3* mutants (Rogers and Guerinot, 2002). Finally, we incorporated the observation that excess amounts of Zn trigger the elimination of Zn from the root symplast by means of PCR2 (Song et al., 2010).

We created two versions of the model, one including IREG1 and the other without it. A comparison of simulation results allowed us to predict the importance of IREG1 on Fe import. Namely, even if IREG1 is indeed present, it does not appear to contribute significantly to Fe loading into the xylem. Specifically, in a simulation modeling the WBC19 mutant under Kan inhibition, the Fe-Ci transporter is not active, so that IREG1 is the only transporter enabling Fe loading. Yet, under this scenario there is virtually no Fe uptake, suggesting that IREG1 does not significantly contribute to Fe transport. For further analysis, see Section 3.1.3.

#### 2.2.2 Translation of the conceptual into a fully parameterized model

Once a diagram (as in **Figure 1**) has been established that displays the connectivity structure among variables, pathways and regulatory signals that are alleged to be important, it is rather straightforward to construct a symbolic mathematical model. Here, ‘symbolic’ means that all processes depicted in the diagram are included in the model structure, but that no specific functional or numerical representations have been chosen yet. In a second step, specific mathematical functions must be chosen to represent each process, while in a third step these functions are parameterized from data. Details of this phased modeling process for the *Arabidopsis* metal uptake pathway are provided in the **Supplementary Material** and in a Read-Me file on GitHub, accompanying our code.

Many choices are available for mathematical functions representing the processes in the system (Voit, 2017a). As detailed in the **Supplementary Material**, we selected a mixed mass-action and generalized mass action (GMA) format. The construction of such a model from a diagram as in **Figure 1** is straightforward and has been documented generically many times (*e.g.*, Voit, 2013; Voit, 2017b). In a nutshell, every dependent variable is represented by a differential equation and every process affecting this variable is represented as a univariate or multivariate power-law function within this differential equation.

To convert the symbolic GMA model into a numerical model, all parameters must be assigned numerical values in such a manner that they render a good model fit to the available data. This estimation is always a substantial challenge, as available data are usually scarce, and a model of a moderately realistic system requires many parameters. In our case, most processes in the system represent transport steps or the assembly or disassembly of complexes of metals with chelators, which renders it reasonable to choose first-order processes with direct proportionality, corresponding to the mass-action format (Voit et al, 2015). Exceptions are inhibition and activation signals, which are known to have powers with moderately small negative or positive values, respectively (see Ch. 5 of). Nothing definite is known about the turnover rates except that they must be non-negative.

The specification of parameter values occurred for our model in a multi-step process involving several rounds of Monte-Carlo simulations (Stevens, 2022) and manual refinements (see **Supplementary Material**). Ultimately, it is not our goal to identify a single, “optimal” set of parameter values with several decimal places. Instead, given the sparsity of data, the well-known issue of non-identifiability (Gutenkunst et al., 2007; Raue et al., 2009; Srinath and Gunawan, 2010) and the fact that biological systems must be robust and function even if parameters are not optimal, our goal is to estimate “order-of-magnitude values” for all parameters from our various datasets. As shown in the **Supplementary Material**, we tested the robustness of the model with the identified values through sensitivity analyses and further Monte-Carlo simulations, confirming that the model structure fits all data quite well, and that the numerical instantiation of the model is robust within an ensemble of well-fitting models.

Taken together, by using a slightly extended mass action formulation, which is arguably the simplest feasible representation for the processes in our system and the effects of the antibiotic and of mutations, the model structure is quite robust, and its implementation is representative of the metal uptake system and its dynamics in the plant. Importantly, it allows us to perform simulations of various perturbations of the system away from normalcy and to reverse-engineer—and thereby explain—feasible strategies with which the plant may alter the activities of transport processes and the association or disassociation of complexes in order to compensate for deficiencies caused by mutations and the inhibition by Kan.

## 3 Results

### 3.1 Experimental insights

To permit quantitative and qualitative analyses of the metal uptake system, several series of experiments were conducted to generate time courses of metal uptake and analyze the sensitivity to Kan in *frd3*, *wbc19* and *ireg1* mutants.

#### 3.1.1 Metal uptake in control and wbc19 mutants

**Figure 3:**
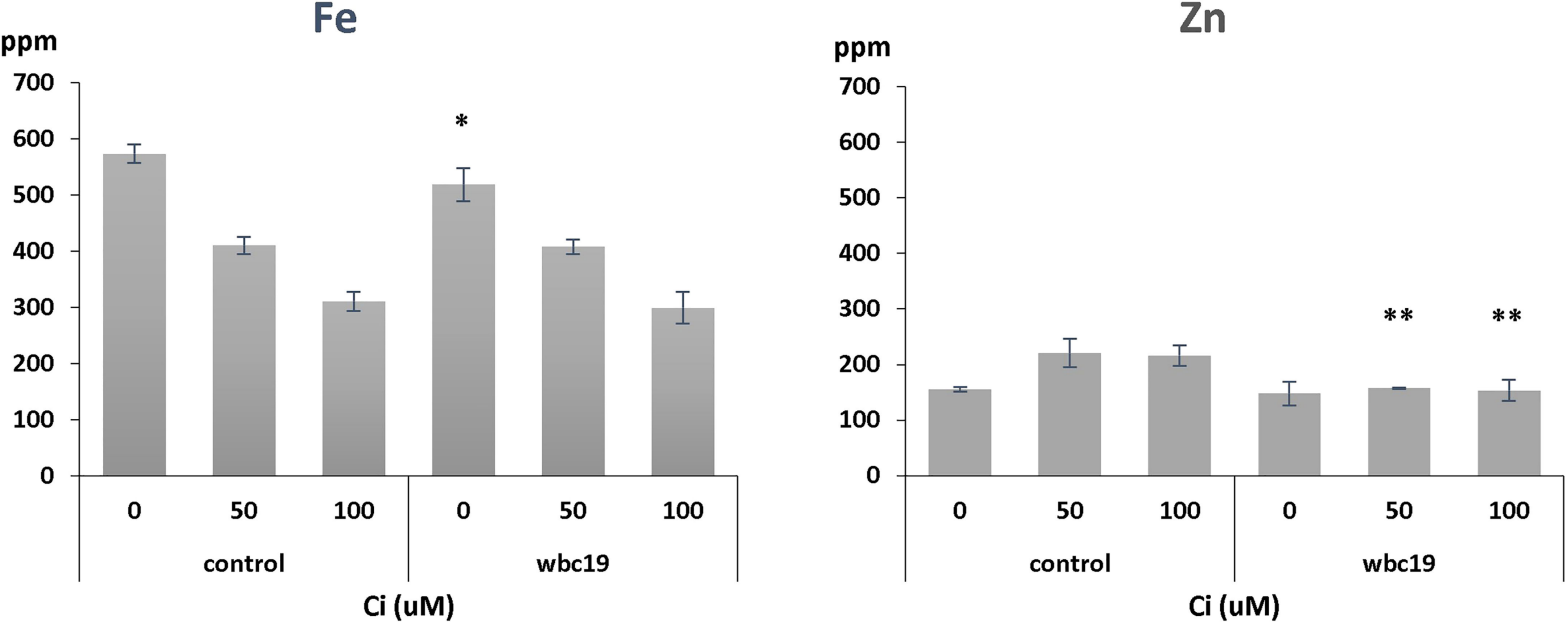
Iron and zinc concentrations in control and *wbc19* mutants grown for 10 days on MS medium supplemented with 0, 50,100 μM Ci. ppm = part per million. Error bars represent standard deviation. * and ** indicate significantly different (p<0.05 and p<0.01 respectively) from control under the same experimental conditions (Student *t*-test).

**Figure 4:**
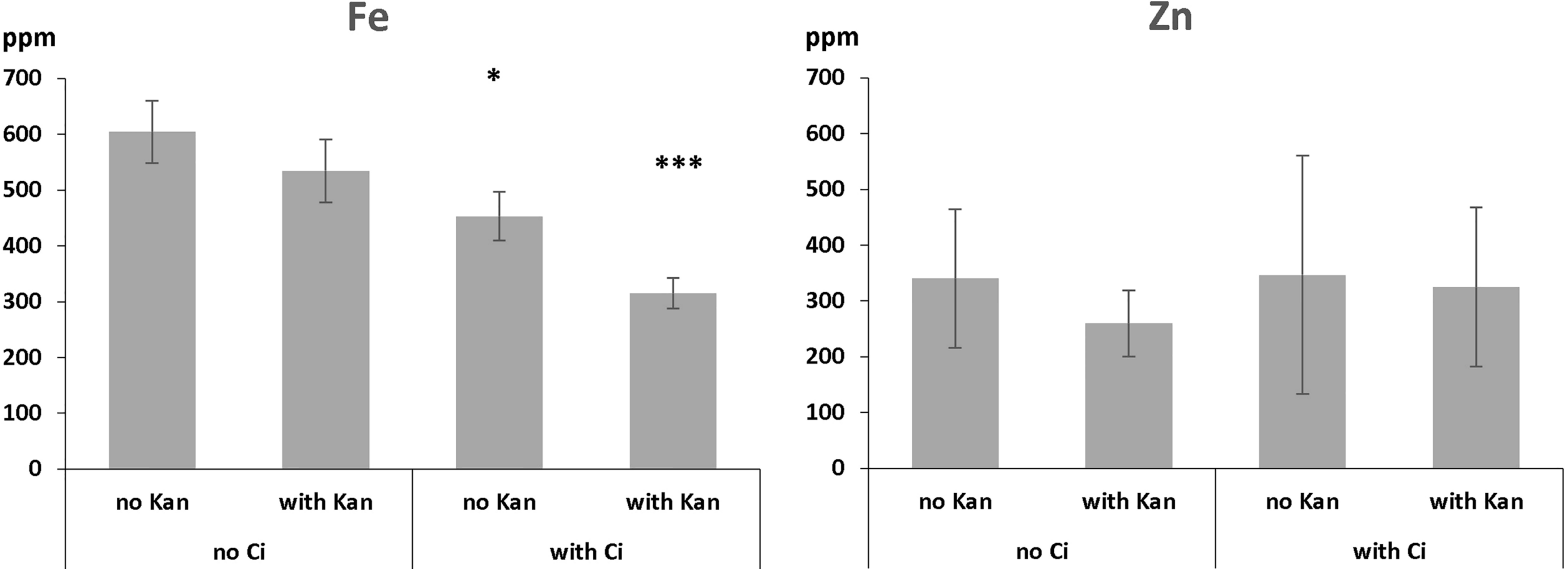
Iron and zinc concentrations in 10-days old control plants as affected by Ci, Kan and a combination thereof. ppm = part per million. Error bars represent standard deviation. * and *** indicate significantly different (p<0.05 and p<0.001 respectively) from control grown on medium without Ci and Kan (Student *t*-test).

Previous experiments indicated that *wbc19* mutants displayed a phenotype in the absence of Kan. Specifically, their uptake of Zn and Cu was much less than controls (Mentewab et al., 2014). However, when experiments were conducted with MS medium prepared in the lab rather than media that are commercially available, the outcomes were found to be different. Total amounts of Fe and Zn taken up are essentially the same for control and *wbc19* mutants at Days 7 and 10 (**Figure 2**). This surprising observation prompted us to examine the composition of the commercially available medium which was found to contain 50µM Ci. Upon addition of Ci to our medium, we were able to recreate this difference in Zn uptake between the WT and the *wbc19* mutant (**Figure 3**). Similarly, exposure to Kan does not strongly alter the Fe and Zn concentrations in the control, but drastically reduces them in the *wbc19* mutant. Therefore, we also examined the simultaneous effect of Ci and Kan on control plants and observed an additive effect between the two on Fe uptake (**Figure 4**). Given these findings, the data generated in the absence of Ci were used as our baseline for further experiments. It is unclear how Ci affects metal homeostasis, although it is apparent that Fe uptake declines with increasing Ci concentrations (**Figure 3**). Ci could chelate metals in the medium and their availability could thus be diminished. Another possibility is for Ci to enter in root cells and bind to metals within and affect their bioavailability and distribution. In either case, the fact that Ci differentially affects the WT and the *wbc19* mutant was instrumental in revealing important features of the mutants. The data displayed in these Figures were used for the parameterization of the computational model (see later).

#### 3.1.2 Effect of Kan on frd3 mutants

**Figure 5:**
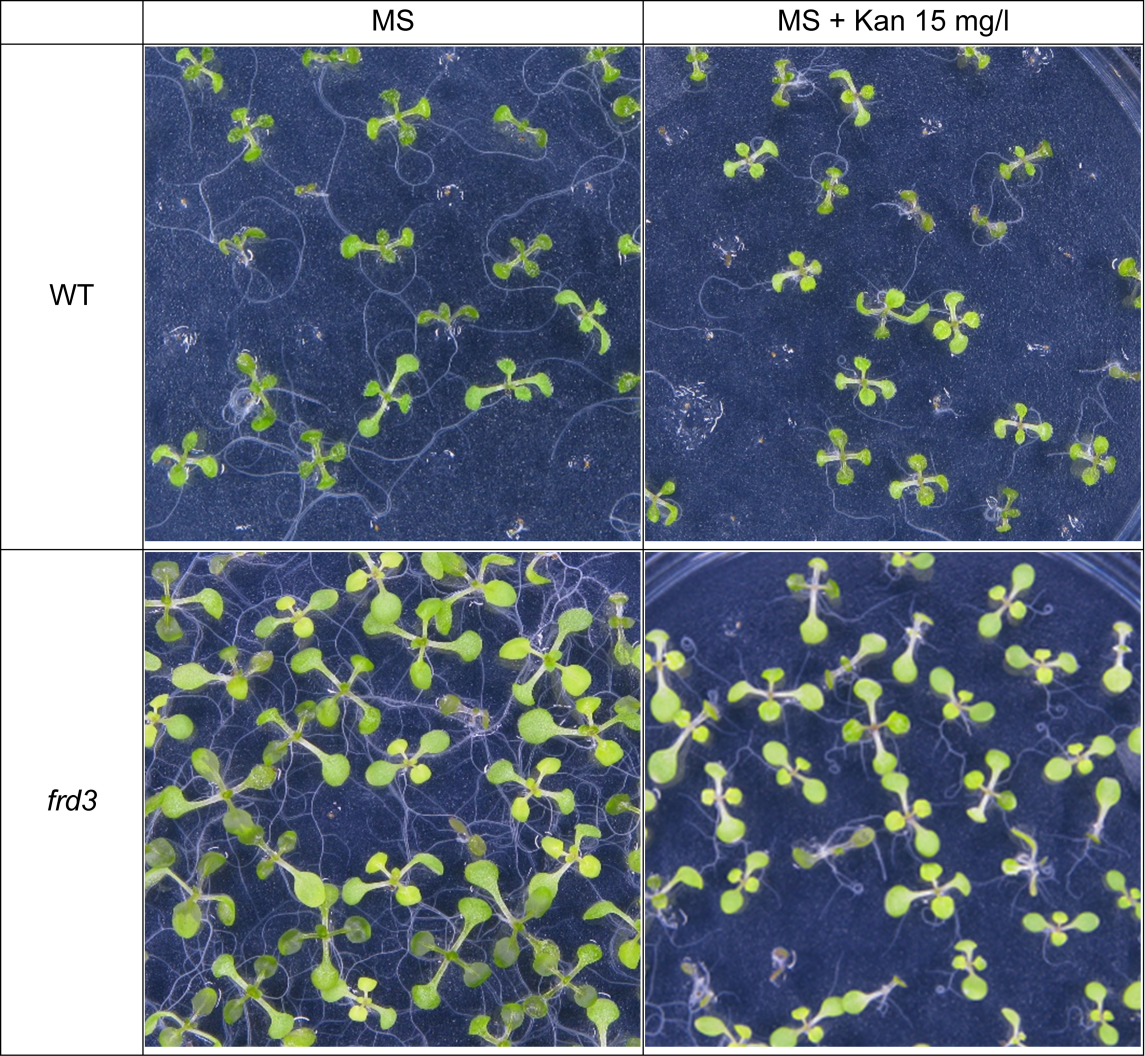
Growth of WT Arabidopsis seedlings and *frd3* mutants on MS medium with or without Kan (15mg/l) for 10 days.

**Figure 6:**
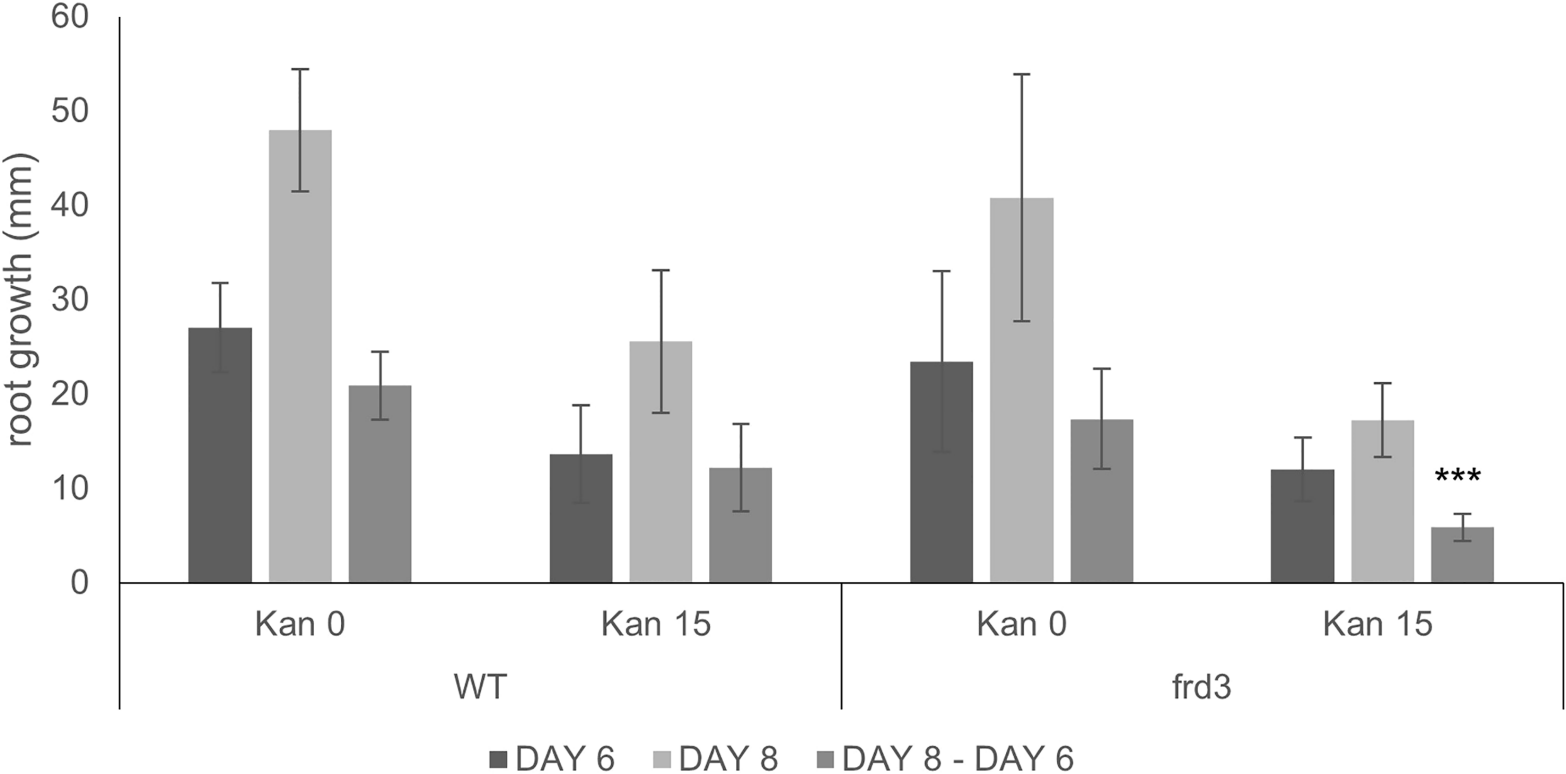
Root growth of WT and *frd3* mutants grown vertically on MS medium with and without Kan 15 mg/l. Root growth was recorded at days 6 and 8 and growth difference between the two dates was calculated. *** denotes P<0.001 when compared to root growth difference for WT on Kan 15 (Student *t*-test).

It is widely accepted that FRD3 transports Ci into the xylem (Durrett et al., 2007). Although Ci levels are lower in *frd3* mutants, with 40% less Ci than in xylem of WT plants, it is not completely missing. Thus, the existence of another Ci transporter is presumed. Initial examination of FRD3 paralogs was not fruitful as mutants of FRD3 paralogues did not exhibit the characteristic phenotype, such as low levels of Ci and iron in xylem sap, accumulation of iron in root vasculature, chlorosis and constitutive iron deficiency responses (Durrett et al., 2007). Further, in crosses with *frd3* mutants, they also failed to exacerbate the *frd3* mutant phenotype. Thus, a transporter other than the FRD3 paralogs likely enables the movement of Ci into the xylem. Our conceptual and computational models provide for this additional transport of Ci, in the form of Fe-Ci complexes. Given that the Fe-Ci transporter in our model is inhibited by Kan, we predict that the phenotype of the *frd3* mutant would be exacerbated by exposure to Kan. These predictions were supported when we examined the phenotype and metal uptake pattern of *frd3* mutants. Under standard conditions, *frd3* mutants displayed their characteristic slightly chlorotic phenotype. In the presence of Kan, they exhibited more uniform chlorosis and impaired root growth, particularly evident in terms of growth rate between days 6 and 8 (**Figures 5**, **6**).

**Figure 7:**
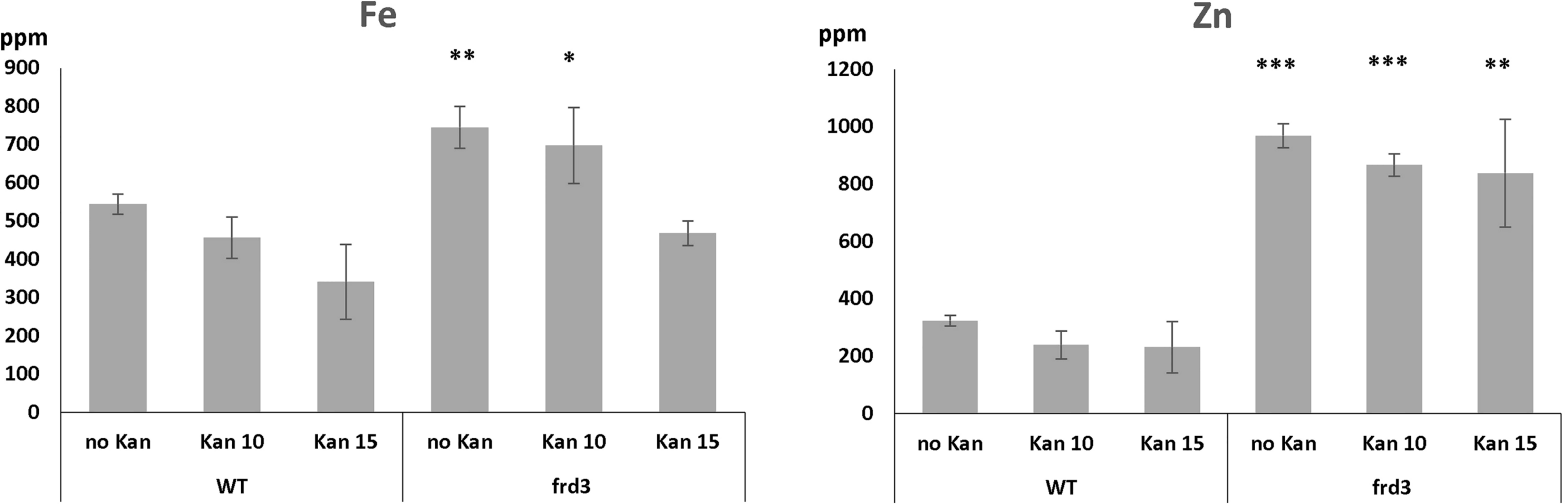
Iron and zinc concentrations in WT and *frd3* mutants grown for 10 days on MS with 0,10 or 15 mg/l Kan. ppm = part per million. Error bars represent standard deviation. *, ** and *** indicate significantly different (*p*<0.05, *p*<0.01 and *p*<0.001 respectively) from WT control grown under the same condition (Student *t*-test).

Upon metal analysis, *frd3* mutants displayed their characteristic high levels of Fe, both when they were grown in the presence or absence of Kan (**Figure 7**). This Fe accumulation is attributed to the fact that low levels of Ci in the xylem result in precipitation of Fe in xylem and preclude efficient Fe mobilization and bioavailability (Green and Rogers, 2004). Ultimately, the consequence is an Fe deficiency response, even when plants are grown under iron sufficient conditions. This response includes an increased Fe(III) chelate reductase activity, and an increased expression of IRT1. Given that IRT1 mediates the uptake of Fe, and other bivalent metals such as Zn and Mn, the *frd3* mutation results in plants accumulating these metals in their shoots (Yi and Guerinot, 1996; Rogers and Guerinot, 2002). Our results (**Figure 7**) also show high levels of Zn accumulation in *frd3* mutants.

#### 3.1.3 Effect of Kan on Ireg1 mutants

**Figure 8:**
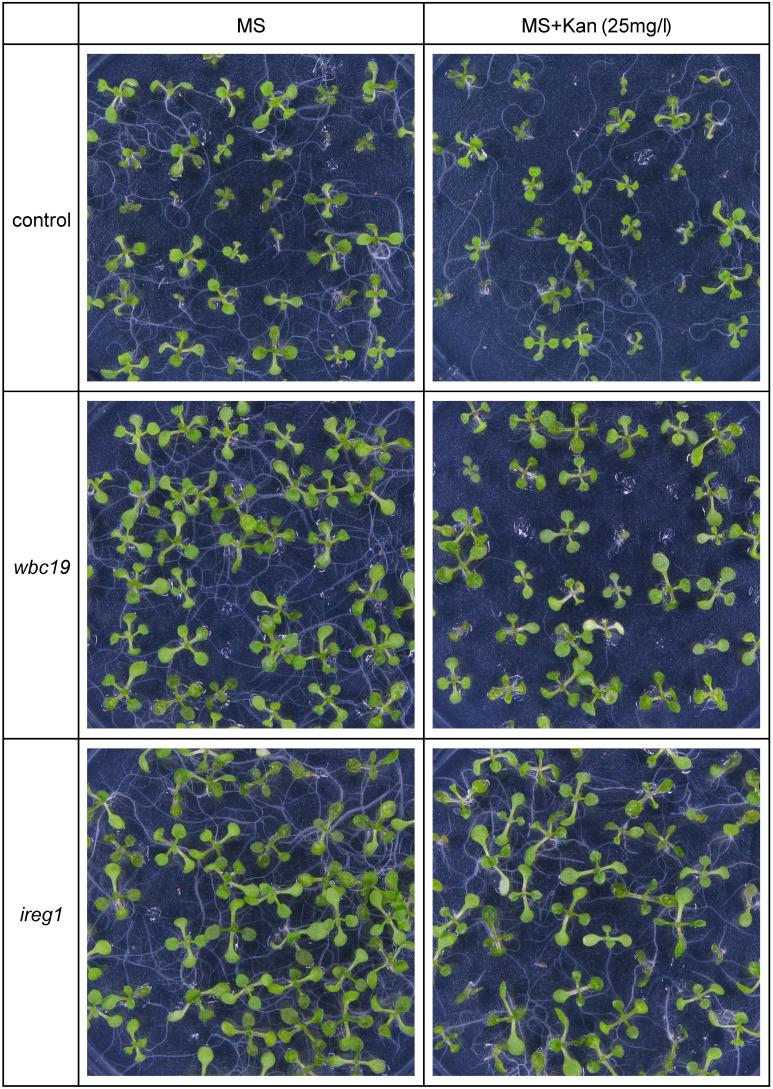
Growth of 10-days old control, *wbc19* and *ireg1* Arabidopsis seedlings on media with 0 or 25 mg/l Kan.

**Figure 9:**
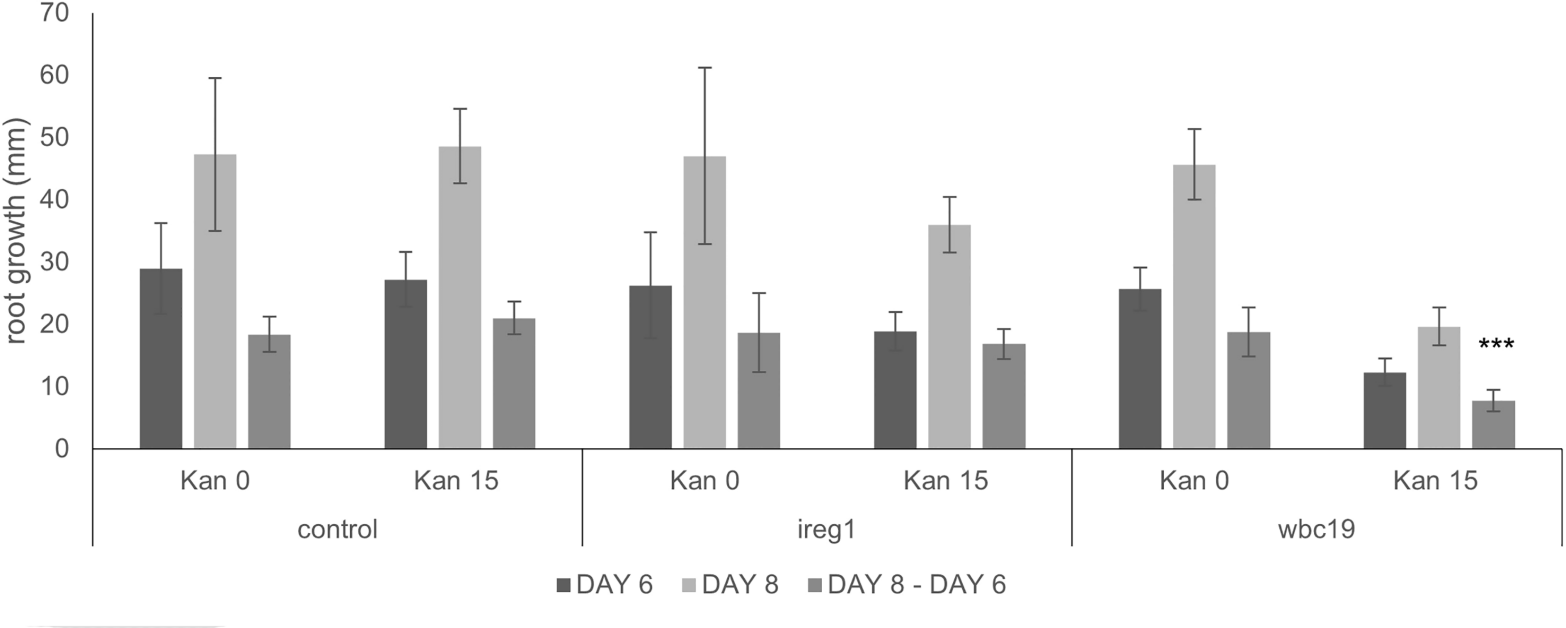
Root length of control, *ireg1* and *wbc19* mutants grown vertically on MS medium with and without Kan 15 mg/l. Root growth was recorded at days 6 and 8 and growth difference between the two dates was calculated. *** denotes *p*<0.001 when compared to root growth difference when no Kan (Student *t*-test).

Based on its similarity to IREG2, which was shown to transport Fe and Co, IREG1 is viewed as one of the major Fe transporters responsible for free Fe transport into the xylem (Morrissey et al., 2009). Further, *ireg1* mutants were found to have low levels of Co in shoots but their Fe content was normal. This observation led to the hypothesis that another Fe transporter might exist, which would have a low or no affinity for Co (Morrissey et al., 2009). Using the model, we performed simulations with and without IREG1. These demonstrated that the role of IREG1 would be minor if it indeed served as a transporter of Fe into the xylem (see Section 2.2.1). To assess the situation further, we examined the growth and metal content profile of *ireg1* mutants exposed to Kan. As previously reported, *ireg1* mutants did not show any distinct visual phenotype under normal conditions (**Figure 8**). During exposure to Kan, *ireg1* mutants were much less sensitive to Kan than *wbc19* mutants. This is particularly apparent during root growth between days 6 to 8, with *wbc19* mutants exhibiting much less root growth on media with Kan 15mg/l (**Figure 9**). These findings indicated that IREG1 is not a critical component of Fe transport during exposure to Kan, and this conclusion is corroborated by model simulations. We also considered the possibility that IREG1 could be the Fe-Ci transporter inhibited by Kan. However, under this scenario, *ireg1* mutants would be highly resistant to Kan, and their metal uptake would not be affected by Kan, which was not the case. Given these observed outcomes, IREG1 was no longer considered in the model.

### 3.2 Features of the computational model, insights and predictions

We used our *de novo* generated data to construct and parameterize a regulated compartment model (**Figure 1**) that captures the changes in Fe and Zn in the wildtype plant biomass well (**Table 2**; **Figure 2**); details of the model design process are described in the **Supplementary Material**.

**Table 2:** Observed metal values (± SD) and values produced by the model, in units of μg of metal taken up by plants grown in one plate.

Scenario	Fe @ Day 10		Fe @ Day 13		Zn @ Day 10		Zn @ Day 13	
	Obs.	Model	Obs.	Model	Obs.	Model	Obs.	Model
**Control**	9.79 ± 1.35	9.95	19.13 ± 2.68	19.18	2.90 ± 0.82	2.81	18.65 ± 3.17	18.34
***wbc19* mutant**	10.32 ± 0.63	9.95	20.10 ± 0.30	20.30	2.03 ± 0.01	2.38	9.58 ± 0.81	9.44
**Kan inhibition**	6.99 ± 2.08	7.27	17.99 ± 2.54	17.38	1.94 ± 0.38	1.52	13.45 ± 2.61	13.62
***wbc19* mutant + Kan**	1.95 ± 0.09	1.87	5.11 ± 0.70	5.22	1.65 ± 0.18	1.01	3.58 ± 0.24	3.64

#### 3.2.1 General model features

Supposing that the diagram of the metal uptake system in **Figure 1** is appropriate, the extended mass-action structure of the model is arguably the simplest viable option. Of course, the model could easily be made more complicated, both in terms of structure and process representations, but our particular model choice seems justified, because the fluxes consist of transport steps among compartments and mechanisms of complex assembly and disassembly, for which the first-order mass-action format is well-suited (Jacquez, 1996; Voit et al., 2015). Furthermore, all inhibitory signals are represented in the simplest possible format, and no assumptions regarding specific inhibitory mechanisms are needed (Voit, 2013; Voit, 2017a).

Several parameters are rather insensitive, and their values can be varied considerably without affecting the data fits (see **Supplementary Material**). Prime examples are the parameters representing transport within and out of xylem. This insensitivity is related to the fact that the model accounts for the total amounts of all Fe- and Zn-compounds in xylem and post-xylem biomass and that biomass is represented as a single compartment where metals are collected.

As discussed in the **Supplementary Material**, other combinations of parameter values yield good results as well. However, supposing that the diagram in **Figure 1** adequately reflects reality, and given that the model captures all available datasets quite accurately, the chosen parameter values appear to be at least coarsely representative of the comparative analyses we perform with the regulated compartment system and, in particular, for the exploration of compensatory processes.

The model is rich enough to account for all experimental scenarios tested, with or without Kan, and in *wbc19* or *frd3* mutants. The various scenarios are implemented by adjusting some of the parameter values in a compensatory manner, while leaving the common model structure itself unchanged. The rationale for this type of analysis is that it appears to be almost certain that *Arabidopsis* responds physiologically to Kan inhibition or to mutated genes in some fashion. However, it is not at all clear which specific pathways the plant alters and to what degree. It is thus an important purpose of our model analysis to explore feasible compensatory strategies.

For our specific model structure, the assessment of parameter adjustments is entirely transparent, due to the choice of mass action and power-law functions, where every parameter has a uniquely defined biological meaning, either as a rate constant or, in the case of kinetic orders, as the direct effect of a variable in question on a specific flux. The results demonstrate that the same model structure with biologically reasonable parameter adjustments is capable of matching the experimental data for all scenarios very well.

While we can readily demonstrate that some sets of parameter adjustments yield effective compensation, it is not possible to establish a complete domain of all feasible parameter combinations for a given situation. There is no analytical method to accomplish this task and a simulation study would require the comprehensive testing of possible changes in unknown magnitudes for some or all parameter values. For static metabolic systems, a constrained optimization method, *Minimization of Metabolic Adjustment* (MOMA; Segrè et al., 2002), was developed to determine which changes in fluxes could move an altered system as close to normalcy as possible. We cannot apply this method here, as the plant is growing. We therefore use the observation data from our experiments, as described above, to reverse-engineer efficacious, biologically feasible adjustments in process activities, with which the plant tries to compensate for reduced transport due to *wbc19* and *frd3* mutations or Kan inhibition.

Although one needs to be cautious with interpretations of specific parameter values, due to the non-uniqueness of solutions, three trends emerge. First, among the transport processes into the xylem (see **Figure 1**), FRD3 has a higher flux than WBC19 (V4_9 > V5_11), which has higher flux than the unknown Fe-Ci transport (V3_8); An effective specific setting is V4_9 = 10; V5_11 = 2; V3_8 = 1. Second, the reversible reactions within the root symplast have a strongly preferred reaction toward xylem and biomass: Chelation of Fe with CI (V1_3) is 30 times as active as disassociation (V3_1); chelation of Fe with NA (V1_5) is 20 times as active as disassociation (V5_1); and chelation of Zn with NA (V2_ 7) is 4 times as active as disassociation (V7_2). Third, the reversible reactions in the xylem point toward Fe-Ci: chelation in the xylem (V9_8) is 2.5 times as active as disassociation (V8_9); disassociation of Fe-NA (V11_10) is 40 times as active as chelation (V10_11). Finally, the precipitation of Fe in xylem (V10_10p) is almost negligible in the wildtype, although it is more pronounced in *frd3* mutations.

#### 3.2.2 Exploration of compensatory strategies

If an organism is mutated or if the magnitudes of any important internal processes are altered by external forces, the organism typically attempts to compensate for the “damage” by up- or down-regulating genes, enzymes or other drivers of the system; for a beautiful example see Ishii et al. (2007). This type of compensation is evidently happening in *Arabidopsis*’ metal uptake system as well, as the *wbc19* mutation would be expected to cause reduced Fe in biomass which, however, is not observed. Similarly, Kan inhibition, which drastically reduces Fe-Ci transport into the xylem, would be expected to have a substantial effect on biomass Fe, but does not (**Figure 2**). Whereas it is thus quite evident that the plant compensates, the mechanisms of this compensation are *a priori* unknown and not easy to predict intuitively. An important purpose of our model is therefore to explore candidate mechanisms for the observed types of compensation.

The overall result of our analysis is that the regulated compartment model, like the actual plant, is capable of making reasonable adjustments in processes other than the dysfunctional process themselves, in order to compensate for the *wbc19* mutation and Kan inhibition. The model also captures the essence of the system with *frd3* mutation (see later section). At the same time, the model does not permit compensation for the combination of the two, like it was observed *in vivo*, and both the actual and virtual plants are deficient in biomass Fe and Zn (**Figure 2**).

Not surprisingly, the compensatory strategies inferred in the following are somewhat *ad hoc* and certainly not unique, as discussed before. Importantly, however, the proposed type of model analysis allows an investigator interested in this topic to test essentially any hypotheses pertaining to possible adaptations.

The parameter adjustments needed to match our experimental results may involve metal uptake and/or changes in specific transport processes. Some of these “solution strategies” are easy to rationalize, while others are not. Also, some strategies are mathematically feasible, but not biologically realistic. It is interesting to note that the actually observed compensation in *Arabidopsis* is not always complete. For instance, Fe in biomass is fully recovered in the *wbc19* mutation, while Zn reaches only about half the wildtype level at Day 13. Intriguingly, the model demonstrates that full compensation could easily be reached with slight adjustments of the strategies described below. This discrepancy between wildtype and mutated system may suggest that the “optimal” amount of Fe is more important to the plant than the amount of Zn. Indeed, observations demonstrate that the plant tries to increase Fe uptake during Fe deficiency and ends up taking up many other metals, especially Zn. The model results also seem to indicate that metal uptake in *wbc19* mutants is dictated by Fe homeostasis, which apparently overrules Zn homeostasis.

***Compensation for wbc19 mutation***. The *wbc19* mutation is readily implemented in the model by eliminating process V5_11 which, by means of WBC19, transports the Fe-NA complex from the root symplast into xylem (*cf.* **Figure 1**). The result of this mutation, without compensation, would be vastly reduced Fe and Zn in the biomass (**Table 3**). The plant may compensate for the lack of WBC19 transport with distinctly different strategies, which are not always intuitive. Some involve changes in metal uptake, while others involve increases or decreases in transport steps within the plant.

**Table 3:** Fe and Zn in biomass of the wbc19 mutant as observed (± SD) and in the model without and with compensation (as depicted in **Figure 2**).

Day	Metal	Observed	Model without Compensation	Model with Compensation
**10**	**Fe**	10.32 ± 0.63	7.78	9.95
	**Zn**	2.03 ± 0.01	2.42	2.37
**13**	**Fe**	20.10 ± 0.30	9.36	20.30
	**Zn**	9.58 ± 0.81	4.22	9.43

Units are μg of metal taken up by plants grown in one plate.

***Compensatory Alternative 1:*** A strong increase in Fe uptake (VS1), while reducing the uptake of Zn (VS2) and generation of Ci (VS4), is sufficient. Specifically, setting VS1 × 8, VS2 × 0.3 and VS4 × 0.5 leads to the results in **Table 3** and **Figure 2**. While mathematically feasible, this strategy appears to be unrealistic, as VS1 and VS2 should operate at the same order of magnitude because both are taken up per the IRT system. As an aside, slightly modifying these settings and defining instead VS1 × 7, VS2 × 0.8 and VS4 × 0.5 would essentially lead to full compensation: At Day 13, the result would be Fe = 20.23, Zn = 19.77, which is very close to observed wildtype values. However, this outcome is not what we observe in the plant, which suggests that Zn is only of secondary importance to the plant.

***Compensatory Alternative 2:*** Moderately increasing uptake of Fe and Zn (VS1 and VS2) and decreasing the transport of Zn into xylem (V2_14), by setting VS1 × 1.4, VS2 × 2.2 and V2_14 × 0.5, leads to results very similar to those in **Table 3** and **Figure 2**.

***Compensatory Alternative 3:*** Moderately increasing the uptake of just Zn (VS2) and decreasing transport of Zn into xylem (V2_14) leads to similar results as before; specifically: VS2 × 4.6 and V2_14 × 0.4.

***Compensatory Alternative 4:*** The plant could not change Fe or Zn uptake at all, but increase Fe chelation with Ci (V1_3), as well as Fe-Ci transport into the xylem (V3_8), and decrease transport of Zn into xylem (V2_14). Specifically, setting V1_3 × 2.5, V3_8 × 2.5, and V2_14 × 0.3 leads to similar results as in **Table 3**. Modifying V2_14 slightly stronger would lead to full compensation (*e.g.*, V1_3 × 2.5, V3_8 × 2.5, and V2_14 × 1.6).

The model demonstrates that the elimination of Fe-NA transport, caused by the missing transporter WBC19, can be ameliorated, and indeed fully compensated, in distinctly different ways. Among the four strategies listed, which are mathematically yielding essentially the same outcome in terms of Fe and Zn in biomass, Alternative 4 might be the most realistic in biological terms. This strategy could be the result of increased synthesis of Ci as stated above and/or a decrease of NA synthesis in the root cells. Indeed, it seems plausible that NA or NA-metals might accumulate and block further synthesis of NA, if they are not removed *via* WBC19. Furthermore, increased uptake of Fe in *wbc19* mutants seems unlikely. As plants grow, there may be a need to increase Fe uptake *via* IRT1, which could result in a gradual increase of Zn uptake in *wbc19* mutants.

Many other, slightly modified or quite different, targeted adjustments of rates could be identified as successful. At the same time, it is important to note that the vast majority of randomly chosen settings would lead to very different outcomes. In fact, essentially all random changes in parameter values lead to enormous differences between observations and model results (see **Supplementary Material**). Thus, a few “ensembles of effective strategies” are possible within a huge domain of counterproductive settings. These strategies offer new, experimentally testable hypotheses. Namely, they suggest measuring specific uptake and flux rates, which would identify those compensatory mechanisms the plant actually uses.

The successful alternatives discussed above were intentionally designed to involve moderate changes in small numbers of parameter values. It is well possible, and indeed likely, that *Arabidopsis* does not use any of these directly but combines them, involving a much larger number of parameter adjustments, each with smaller magnitude than what is described above. As an analogy, it was observed that yeast cells under diauxic stress launch a genetic program where many genes are slightly altered in expression, even though a few large changes would have the same overall effect (Alvarez-Vasquez et al., 2007). The reason for a broadly distributed response is speculated to be that side effects are presumably much milder under many small changes than under a few large changes (Lee et al., 2011).

***Compensation for Inhibition by Kan.*** Kan strongly inhibits the important transport of the Fe-Ci complex into xylem. We supposed that 10% activity of the process is left under inhibition. As in the case of mutated *wcb19*, both Fe and Zn would be drastically reduced without compensation (**Table 4**). The reduction in Fe transport into the xylem due to Kan can again be compensated by the plant in different ways.

**Table 4:** Fe and Zn in biomass of *Arabidopsis* under Kan exposure as observed (± SD) and in the model without and with compensation (as depicted in **Figure 2**).

Day	Metal	Observed	Model without Compensation	Model with Compensation
**10**	**Fe**	6.99 ± 2.08	5.45	7.27
	**Zn**	1.94 ± 0.38	1.46	1.52
**13**	**Fe**	17.99 ± 2.54	7.53	17.38
	**Zn**	13.45 ± 2.61	1.72	13.62

Units are μg of metal taken up by plants grown in one plate.

***Compensatory Alternative 1:*** Intriguingly, a strong increase in uptake of Fe (VS1) alone would be sufficient to compensate for the inhibition by Kan: VS1 × 4.4 leads to the results in **Table 4** and **Figure 2**. While mathematically very simple and effective, this alternative is not realistic though as a strong increase in Fe uptake would be accompanied by a similar increase in Zn uptake, as both are taken up per the IRT system. However, an increased amount of Zn is not observed in plants exposed to Kan.

***Compensatory Alternative 2:*** A more likely scenario is a moderate increase in uptake of Fe (VS1), combined with rechanneling most of Fe toward the chelator NA (V1_5 and V5_11). Specifically, VS1 × 2.75, V1_5 × 2, V5_11 × 2 results in effective compensation, yielding model values close to the observation.

***Compensatory Alternative 3:*** Mathematically, an increase in the uptake of Fe is not even needed. Instead, Fe could be channeled toward chelation with NA rather than CI (V1_5 combined with V3_1 and V5_11), while Ci transport into xylem would be increased (V4_9). Specifically, setting V1_5 × 25, V3_1 × 25, V4_9 × 25 and V5_11 × 25 leads to compensation as observed. It is unknown whether such strong upregulation is biologically feasible or likely.

As in the case of WBC19, other scenarios are possible and could create uncounted differently combined strategies. Still, their number would be minute among all possible random changes in parameter values (see **Supplementary Material**).

***Compensation for mutated wbc19, combined with Kan inhibition.*** The combined reduction of the transport of the two complexes Fe-Ci and Fe-NA has dramatic consequences that cannot be corrected with any reasonable adjustments of rates. As the observations attest, only small amounts of Fe and Zn accumulate in biomass in this case, both well below the wildtype levels. The model without compensation yields even lower values, but even compensatory attempts with very strong uptake of Fe, Zn and Ci (VS1 × 102, VS2 × 20, VS4 × 100) would be insufficient to regain normalcy, even though they capture the observation data (**Table 5**). An even more dramatic increase in VS1 (VS1 × 200, VS2 × 10, VS4 × 20) could restore values close to the control but would probably not be biologically plausible. Furthermore, as both Fe and Zn uptake (VS1 and VS2) involve the same transporter (IRT1), the strong differential in uptake would have to come through FRO2, which seems unlikely.

**Table 5:** Fe and Zn in biomass of the *wbc19* mutant under Kan exposure as observed (± SD) and in the model without and with compensation (as depicted in **Figure 2**).

Day	Metal	Observed	Model without Compensation	Model with Compensation
**10**	**Fe**	1.95 ± 0.09	1.47	1.87
	**Zn**	1.65 ± 0.18	0.81	1.01
**13**	**Fe**	5.11 ± 0.70	1.70	5.22
	**Zn**	3.58 ± 0.24	0.83	3.64

Units are μg of metal taken up by plants grown in one plate.

#### 3.2.3 Model Predictions

In addition to insights into compensatory strategies, outcomes of untested scenarios may be tested with the model. As examples, we performed simulations of an *frd3* mutation and of an *frd3/wbc19* double mutation, as well as an *frd3* knockout of under Kan inhibition of the unknown Fe-Ci transporter (**Table 6**).

**Table 6:** Total biomass Fe, precipitated Fe, and Zn in biomass of the *frd3* mutant, the double knockout (*frd3* and *wbc19*), and the *frd3* mutant under Kan exposure at Day 10.

Scenario	Total Amount in Xylem and Post-Xylem Biomass		Precipitated Fe in Xylem
	Fe	Zn	
**Wildtype**	9.95	2.81	0.89
***frd3* mutant**	13.76	5.78	2.02
***frd3*/*wbc19* double mutant**	10.46	3.77	1.47
***frd3* + Kan inhibition**	11.0	3.52	1.74
**Wildtype + Kan inhibition**	7.27	1.52	0.56

Wildtype values are provided for comparisons. Units are μg of metal taken up by plants grown in one plate.

If FRD3 is not functional, much less Ci is available in the xylem. It has also been observed that much Fe precipitates in xylem. Because this precipitated Fe does not enter the cells, the plants are in effect experiencing Fe deficiency and are chlorotic, seen in their yellowish appearance. In response, the plant upregulates Fe uptake from the medium *via* IRT1. As IRT1 is not very selective, and the plants take up not only Fe, but also Zn.

It is straightforward to implement these events into the model: We simply eliminate Ci transport into the xylem, due to the *frd3* mutation (V4_9 = 0; **Figure 1**), remove most of the Ci concentration in xylem at the beginning of the experiment, and estimate initial concentrations from **Figure 7** as Fe = 6.7, Zn = 5.6 at Day 7. These settings lead to increases in both Fe and Zn in biomass. At Day 10, the predicted levels are Fe = 13.76, Zn = 5.78. That is, Fe is about 30% higher and Zn is more than doubled in comparison to wildtype (Fe = 9.95, Zn = 2.81), which is consistent with observations (**Figure 7**). The amount of precipitated Fe in xylem more than doubles, from 0.89 to 2.02 (**Table 6**).

To assess the double mutant (*wbc19* = 0, *frd3* = 0), we start with the compensation for the *wbc19* mutation (described above as Alternative 4) and additionally set FRD3 = 0. We also assume that there is very little Ci in xylem at the beginning of the computational experiment. We do not know the appropriate start values of Fe and Zn for this situation and therefore average them from the values for the *wbc19* and *frd3* mutants. Thus, using Fe = 5.8, Zn = 3.7 at Day 7, the result is a noticeable increase in Fe as well as Zn in biomass in comparison to wildtype; at Day 10, we obtain Fe = 10.46, Zn = 3.77 as opposed to Fe = 9.95, Zn = 2.81 in wildtype (**Table 5**).

Instead of the *wbc19* knockout, we can study the loss of FRD3 function combined with Kan inhibition. Specifically, we implement the simulation again as kan = 0.1, with compensation described above as Alternative 2, and additionally set FRD3 = 0. We again do not know the appropriate start values of Fe and Zn and average them from the value for the system under Kan exposure and the *frd3* mutation (Fe = 4.9, Zn = 3.4 at Day 7). The result at Day 10 (Fe = 11.0, Zn = 3.5) suggests an increase in Fe and Zn in biomass compared to wildtype at Day 10, where Fe = 9.95, Zn = 2.81. The results for Fe are comparable to the values of the wildtype under Kan exposure (Fe = 7.27, Zn = 1.52; **Table 6**; **Figure 7**). However, the model does not suggest as strong an increase in Zn as observed. It is possible that the plant would trigger additional compensatory mechanisms in this situation.

## 4 Discussion

Our quest to understand the relationship between metal homeostasis and the effect of Kan prompted us to take a combined experimental-modeling approach, because it is apparent from the diagram in **Figure 1** that the actions and interactions of the various metals, chelators and transporters can hardly be understood intuitively. To the best of our knowledge, this approach resulted in the first comprehensive model for metal loading and transport in *Arabidopsis*. The proposed compartment model captures the wildtype system well. Importantly, it permitted us to explore compensatory strategies with which the plant might effectively respond to adverse conditions. Many authors in the past have proposed inferences of regulatory signals in particular systems and some have even developed systematic methods to optimize the absence or presence of regulatory signals in a biological system (*e.g.*, Hatzimanikatis et al., 1996a; Hatzimanikatis et al., 1996b). These methods were not feasible here, because we were interested in magnitudes of changes, not just presence or absence, and because the plants are not static and, in particular, grow during the experiment. While the strategies we inferred and documented here were non-unique and *ad hoc*, we were able to demonstrate how one may use a model of this type to test novel hypotheses regarding potentially effective strategies compensating for adverse situations.

The model adds two components to our previous understanding of long-distance Fe transport. These are an unknown Fe-Ci transporter and WBC19 as an Fe-NA transporter (and possibly other metal-NA or NA transporter). Together with FRD3, which was previously established as a Ci transporter, the model captures the interplay between the substrates as they move among the different compartments by means of the three transporters. The model exhibits some degree of natural redundancy, with two of the transporters enabling the transport of Fe and two enabling the transport of Ci. Yet, each transporter allows the movement of different forms of Fe and chelators, namely Fe-Ci, Fe-NA and Ci, but none of these are fully equivalent. Furthermore, in addition to Fe, these transporters may also transport other metal chelates. Our model does not attempt to specify the speciation of the various metal-chelator complexes as these are dynamic and there is a great degree of uncertainty as to which specific forms are transported. Overall, metal movement through each of these transporters will be dictated by their presence, activity, and affinity for their substrates and the abundance of their respective substrates.

As a proof of concept—and possibly the starting point for more sophisticated analyses—our computational model only considers Fe and Zn. Clearly, the model could be extended toward other metals in a process analogous to the design and parameterization proposed here (see **Supplementary Material**). We focused here on Fe and Zn because these are the two most abundant micronutrients in plants and their affinities to the chelators Ci and NA have been studied through experimentation (von Wiren et al., 1999). However, the details of their transport through a seemingly redundant system, and the regulatory responses of the plant, are not obvious.

In addition to the components of xylem loading, our model integrates known regulatory responses to iron deficiency mediated by local and systemic signals (Vert et al., 2003). The proposed model agrees with our experimental results, both qualitatively and quantitatively, and recapitulates the phenotypes of the *wbc19* and *frd3* mutants under standard conditions and during exposure to Kan. Specifically it captures the distinctive Kan sensitive phenotype of *wbc19* mutants and the increased accumulation of Fe in the xylem and the constitutive iron deficiency response in *frd3* mutants.

An important aspect of the model is the fact that the Fe-Ci transporter is inhibited by Kan. This mechanism explains the strong effect of Kan on *wbc19* mutants and the overall decrease of iron uptake in plants exposed to Kan (Mentewab et al., 2014). In addition, it predicts a strong effect of Kan on *frd3* mutants. This result is corroborated by our experimental results where Kan exacerbates the phenotype of *frd3* mutants. The potential mechanism and significance of the inhibition of the Fe-Ci transporter by Kan is unknown. However, one possibility is that *Arabidopsis* plants limit Fe-Ci transport to prevent Kan transport through the same transporter. Indeed, it is thought that aminoglycoside antibiotics bear structural similarities with Fe-chelate complexes enabling them to move through Fe-chelate transporters such as MAR1/IREG3 (Conte and Lloyd, 2010). As a result, when MAR1 is mutated, seedlings exhibit increased resistance to antibiotics (Conte et al., 2009). A similar scenario may be at play with our putative Fe-Ci transporter where inhibition by Kan may represent an adaptive response. If so, the current model would represent a subtle mechanism that allows plants to limit the movement of Kan without excessively impacting plant nutrition.

In a novel twist of using a dynamic model, we used the model to explain how *Arabidopsis* may respond to mutants and to inhibition by Kan. Without compensation, the *wcb19* mutation, for instance, would by itself lead to much lower amounts of Fe and Zn in biomass (**Table 3**). However, that is not what we observe. It would be difficult to determine intuitively how the dysfunction caused by one of the mutations or Kan inhibition could be compensated quantitatively. The model allows us to explore potential mechanisms for such a compensation, and it will be interesting to test some of these alternative compensation strategies in the plant.

In conclusion, we have established a model that accounts for the observed relationships between Kan and metal uptake in *Arabidopsis*. The model permits the testing of hypotheses regarding metal transport as affected by kanamycin and various mutations. Future work will focus on the identification of the unknown Fe-Ci transporter. The model predicts that mutation of the Fe-Ci transporter would have limited effect on Fe uptake as WBC19 would become an alternate route for Fe transport. However, in double mutants with FRD3, the transport of any free or chelated Ci into the xylem is abrogated, which should result in a phenotype worse than that of *frd3* mutants, provided that such seedlings are viable. Likewise, in double mutants with WBC19, Fe transport into the xylem would be virtually halted which would result in strong Fe deficiency if viable seedlings were generated. By contrast, double mutants with IREG1 should not exhibit a strong phenotype. The model can further be extended to include other metals. Given that Ci and NA chelate a range of metals, components of the model will intersect with the homeostasis of metals such as Cu and Co.

## Data availability statement

The original contributions presented in the study are included in the article/**Supplementary Material**. Further inquiries can be directed to the corresponding author.

## Author contributions

AM and EV conceptualized the model, drafted and reviewed the manuscript. BM, CR, NT-P, TV and LO designed and executed experiments to support the model with guidance from AM. reviewed draft manuscript. CK and EV developed the mathematical and the parametrized computational model and reviewed the manuscript. All authors contributed to the article and approved the submitted version.
