# Supplementary material for: A dynamic compartment model for xylem loading and long-distance transport of iron explains the effect of kanamycin on metal uptake in Arabidopsis
Source: Front Plant Sci. 2023 Apr 18;14:1147598. doi: 10.3389/fpls.2023.1147598 (PMC10151686; doi:10.3389/fpls.2023.1147598)
Supplement: Supplementary file 1 [file DataSheet_1.docx]

# Supplementary Material

**Details regarding the translation of a conceptual model into a mathematical model**

The translation of a diagram into a fully computable ordinary differential equation (ODE) model proceeds generically in four steps (*e.g.*, Voit, 2017b).

1. One equation is set up for each dependent variable. The left-hand side is the derivative of this variable, that is, its change over time. For *X*_1_, we write $\frac{dX_{1}}{dt}$ or use the abbreviated, entirely equivalent notation $\dot{X}_{1}$. The right-hand side of each of these equations consists of all fluxes entering and leaving the pool represented by this variable, with positive and negative signs, respectively. Also, an initial value is to be assigned to each variable.

2. All fluxes are represented with specific mathematical functions.

3. These functions are parameterized from data.

4. The parameterized model is to be checked for consistency and parameter sensitivities.

***Symbolic Equations***

For the diagram in Figure 1 of the Text, the symbolic ordinary differential equations, with abbreviations as shown in the diagram, are:

X1' = VS1 − V1_3 + V3_1 − V1_5 + V5_1

X2' = VS2 − V2_7 + V7_2 − V2_14 − V2_0

X3' = V1_3 − V3_1 − V3_8

X4' = VS4 + V3_4 − V4_3 − V4_9

X5' = V1_5 − V5_1 − V5_11

X6' = VS6 + V5_6 − V6_5 + V7_6 − V6_7 − V6_12

X7' = V2_7 − V7_2 − V7_13

X8' = V3_8 − V8_9 + V9_8 − V8_15

X9' = V4_9 + V8_9 − V9_8

X10' = V8_10 − V10_8 − V10_11 + V11_10 − V10_10p

X10p' = V10_10p

X11' = V5_11 + V10_11 − V11_10 − V11_16

X12' = V6_12 + V11_12 − V12_11 − V12_13 + V13_12

X13' = V7_13 + V12_13 − V13_12 − V13_17

X14' = V2_14 + V13_14 − V14_13 − V14_18

X15' = V8_15

X16' = V11_16

X17' = V13_17

X18' = V14_18

In addition to variables listed in Table1, *S*_1_ and *S*_2_ are independent variables denoting Fe and Zn in the medium, respectively, while *S*_4_ and *S*_6_ describe Ci and NA generated in the symplast. These variables have constant values and do not require their own differential equations. The notation of fluxes and transport steps reflects the source and sink of each process: Each reaction, flux or transport step receives a name like V*p*_*q* if it connects two dependent variables with indices *p* and *q*. As an example, the transport of Fe from root symblast (*X*_3_) into xylem (*X*_8_) is denoted as V3_8 (see Figure 1). For uptake fluxes, we use the simplified notation VS1, …, VS4.

Each variable is assigned an initial value, that is, the numerical value with which a simulation starts. For molecular variants of Fe and Zn in the xylem and biomass, we divided the amounts observed at Day 7, which we used as the initial time point, equally among the contributing variables. Not having any information on the remaining initial values, we set their values equal to 1. Thus, for the wildtype model, we defined the following values:

X1 = 1

X2 = 1

X3 = 1

X4 = 1

X5 = 1

X6 = 1

X7 = 1

X8 = 4.9 / 6

X9 = 1

X10 = 4.9 / 6

X10p = 4.9 / 6

X11 = 4.9 / 6

X12 = 1

X13 = 1.9 / 4

X14 = 1.9 / 4

X15 = 4.9 / 6

X16 = 4.9 / 6

X17 = 1.9 / 4

X18 = 1.9 / 4

***Mathematical Format***

The choice of specific mathematical functions for all fluxes is an unsolved problem and somewhat of an art. We almost exclusively use mass-action representations, which we slightly extended in some cases to the Generalized Mass Action (GMA) format. The reasons for this choice are the following. First, mass-action models are the common default for systems mainly consisting of transport and complex association and disassociation processes (Edelstein-Keshet, 1988). GMA models are direct extensions of mass action models but permit the seamless incorporation of regulatory signals and other nonlinearities. Second, GMA models have been “mathematically vetted,” as they are derived directly from Taylor’s general approximation theory for real-valued functions and embedded within the well-established modeling framework of *Biochemical Systems Theory* (Savageau, 1976; Voit, 1991; Voit, 2000; Torres and Voit, 2002; Voit, 2013). Third, it is straightforward to convert a diagram, such as Figure 1 of the Text, consisting of boxes (variables) and arrows (material flow or signals), into an essentially unique GMA model structure (Voit, 2017). Fourth, the GMA format is rich enough to model any nonlinearities, as long as they are differentiable (Savageau and Voit, 1987). Finally, within the context of metabolic pathway systems, comparative analyses with GMA representations and models based on more traditional generalized Michaelis-Menten and Hill functions have demonstrated that both types yield similar results under normal experimental conditions (Curto *et al.*, 1995; Curto *et al.*, 1998; Alvarez-Vasquez *et al.*, 2004). Within the GMA model structure, we used simple mass-action representations for all processes reflecting transport and complex-assembly or disassembly steps and power-law expressions for inhibitory and activating processes.

To construct a GMA model from the diagram of a system, every flux is represented as a product of a non-negative *rate constant* and of all variables that directly affect this process, raised to an exponent, called a *kinetic order* (Voit, 2013). The direction of the effect (positive, negative, zero) is directly mapped onto the kinetic order, while the strength of the effect is reflected in the magnitude of the kinetic order.

**Fig. A1:** Example pathway for setting up GMA equations.

As an illustration, consider the very simple diagram in Figure A1. Here, *Y*_1_ is supplied with material from the outside of the system and *Y*_2_ is subsequently generated from *Y*_1_. *Y*_3_ inhibits this latter process, while *Y*_4_ activates the degradation or consumption of *Y*_2_. The GMA system for this pathway consists of equations for *Y*_1_ and *Y*_2_; *Y*_3_ and *Y*_4_ do affect these equations but are considered constant external influences that do not receive their own equations. The result is:

$\dot{Y}_{1}= a_{1} - a_{12}Y_{1}^{g_{11}}Y_{3}^{g_{13}}$

$\dot{Y}_{2}= a_{12}Y_{1}^{g_{11}}Y_{3}^{g_{13}} - a_{2}Y_{2}^{g_{22}}Y_{4}^{g}$

The parameters $a_{1}$, $a_{12}$ and $a_{2}$ are the (non-negative) rate constants of the associated processes, while the exponents are all positive, except for *g*_13_, which represents the inhibition process and is therefore negative. Note that one of the terms appears in both equations, although with different signs, because it refers to the same flux.

The equations corresponding to the diagram in Figure 1 of the Text are constructed in the same fashion. As a slight variation, we formulate processes subject to mutations such that they become 0 for total knockouts and terms with inhibition signals are formulated such that they become 1 if there is no inhibition. The result is as follows:

***Fluxes:***

======

VS1 = as1 * S1 * (X8 + X10 + X15 + X16)^g1^

V1_3 = a1_3 * X1 * X4

V3_1 = a3_1 * X3

V1_5 = a1_5 * X1 * X6

V5_1 = a5_1 * X5

==========================

VS4 = as4 * (1 + X12/0.4)^g2^

V2_0 = a2_0 * (1+ (X13 + X14 + X17 + X18))^g3^

V2_7 = a2_7 * X2 * X6

V7_2 = a7_2 * X7

V2_14 = a2_14 * X2

==========================

V3_4 = V3_1

V4_3 = V1_3

V3_8 = a3_8 * X3 * kan

==========================

VS6 = as6 * (1+ X9/0.01)^g4^

V4_9 = a4_9 * X4 * frd3

==========================

V5_6 = V5_1

V5_11 = a5_11 * X5 * atwbc19

==========================

VS2 = as2 S2 * (X8 + X10 + X15 + X16)^g1^

V6_5 = V1_5

V6_7 = V2_7

V6_12 = a6_12 * X6 * atwbc19

==========================

V7_6 = V7_2

V7_13 = a7_13 * X7 * atwbc19

==========================

V8_9 = a8_9 * X8

V8_10 = V8_9

V8_15 = a8_15 * X8

==========================

V9_8 = a9_8 * X9* X10

==========================

V10_8 = V9_8

V10_11 = a10_11 * X10 * X12

V10_10p = a10_10p X10

==========================

V11_10 = a11_10 * X11

V11_12 = V11_10

V11_16 = a11_16 * X11

==========================

V12_11 = V10_11

V12_13 = a12_13 * X12 * X14

==========================

V13_12 = a13_12 * X13

V13_14 = V13_12

V13_17 = a13_17 * X13

==========================

V14_13 = V12_13

V14_18 = a14_18 * X14

Like in the illustration example, these definitions involve two major types of parameters: rate constants (indexed a’s) and activation or inhibition parameters (indexed g’s). The indices of the rate constants are identified exactly like the fluxes, namely by the source and target of a reaction; thus, a1_3 is the rate constant of flux V1_3. For influxes, the rate constants are abbreviated like the fluxes themselves, such as as1 for VS1. The activation or inhibition parameters express that the corresponding variable has an activating or inhibiting effect on some flux, as demonstrated before with the two-variable illustration example. Beyond these two types of parameters, the factors *wbc19*, *frd3*, and *kan* represent the absence or presence of the WBC19 or FRD3 mutation or Kan inhibition, respectively. Under wildtype conditions, their values are 1. In the case of a mutation, the setting *wbc19* = 0 or *frd3* = 0, for instance, codes for total suppression of the activities of the corresponding enzyme, while *kan* = 0.1 codes for a remaining activity of 10% of Fe-Ci transport under Kan inhibition.

***Parameter Determination***

To convert the symbolic GMA model into a numerical model, all rate constants (indexed a’s) and activation or inhibition parameters (indexed g’s) must be assigned numerical values in such a manner that the model renders a good fit to the available data. Rate constants may assume any non-negative values. The value of an activation or inhibition parameter expresses how strongly a variable modulates a process: A value close to 0 represents essentially no effect; values in the range of +2 or +4 represent rather strong activation, while values in the range of -2 or -4 represent strong inhibition (Voit, 2000).

We used for the parameter determination our experimental data in units of μg of metal taken up by plants grown in one plate (Table 1 of the Text). Given the complexity of the model and the relative scarcity of data, the parameter values were determined in several steps. A first Monte-Carlo (MC) situation was used to determine coarse ranges for all parameters. These were refined with manual adjustments to lead to the parameter values below.

as1 = 0.0038

as2 = 0.01

as4 = 14

as6 = 20

a1_3= 3

a1_5 = 2

a2_0 = 0.08

a2_7= 4

a2_14= 0.5

a3_1= 0.1

a3_8 = 1

a4_9 = 10

a5_1= 0.1

a5_11= 2

a6_12= 0

a7_13= 0

a7_2 = 1

a8_9 = 1 a8_15 = 1

a9_8 = 2.5

a10_11 = 0.1

a10_10p = 0.1

a11_10 = 4

a11_16 = 0.1

a12_13 = 0.1

a13_12 = 0.1

a13_17 = 0.1

a14_18 = 0.1

g1 = 3

g2 = -0.3

g3 = 2

g4 = -0.3

Two formats of the baseline model are available on Github: One in Matlab and one in PLAS (www.http://enzymology.fc.ul.pt/software/), a very easy-to-use ODE solver. The Github folder also contains code for Monte-Carlo simulations in both formats. A Read-Me file contains comments and additional details.

***Consistency and Parameter Sensitivities***

By formulating the symbolic equations in terms of fluxes, which are defined separately, we are minimizing numerical errors as we are assured that the same amount leaving some variable enters another pool, even under changes in parameter values. Also, this format facilitates direct comparisons with the diagram upon which the model is based.

To assess the sensitivity of the model to variations in parameter values, we began with the above parameter values, reduced and increased each parameter value by 10% and computed the sum of squared differences (SSD) between Fe and Zn in biomass under the baseline conditions and upon simulation with the altered parameter values at timepoint 13, which correspond to the final observation time point (Table A1). As an example, the first SSD in Table A1 was computed as

SSD = (Fe_B_(*t*=13)_0.9*baseline_ - Fe_B_(*t*=13)_baseline_ )^2^

+ (Fe_B_(*t*=13)_1.1*baseline_ - Fe_B_(*t*=13)_baseline_ )^2^

+ (Zn_B_(*t*=13)_0.9*baseline_ - Zn_B_(*t*=13)_baseline_ )^2^

+ (Zn_B_(*t*=13)_1.1*baseline_ - Zn_B_(*t*=13)_baseline_ )^2^

In this formula, Fe_B_(*t*=13)_0.9*baseline_, for example, represents the total amount of Fe in biomass at Day 13, when the value of parameter *as*1, representing the rate of Fe uptake from the medium, is reduced to 90% of its baseline value. The results of this analysis are displayed in Table A1. They are to be interpreted in the following manner. If SSD for a given parameter is large (shaded pink; for example, SSD with respect to the Fe uptake rate constant as1 or the parameter g1, representing the activation of Fe uptake of by biomass), the model is sensitive to changes in the value of this parameter. In other words, if this parameter value is changed slightly, the effect on Fe and Zn in biomass is considerable. Examples of very high sensitivities are the activation parameters g1 and g3 in the fluxes VS1 and VS2. By contrast, a parameter like a8_15, shaded green and representing transport of Fe-Ci from xylem to post-xylem biomass, yields SSD = 0.001, which indicates that it may be altered quite a bit before changes in Fe or Zn biomass become noticeable.

**Table A1: Summary of Parameter Sensitivity Analysis (see Text for details)**

Particularly high / low values of sums of squared differences (SSD) are highlighted in pink / green

| **Parameter** | **Fe in Biomass** | | |  | **Zn in Biomass** | | | **SSD** |
| --- | --- | --- | --- | --- | --- | --- | --- | --- |
|  | **0.9 * baseline**  **value** | **baseline**  **value** | **1.1 * baseline**  **value** |  | **0.9 * baseline**  **value** | **baseline**  **value** | **1.1 * baseline**  **value** |  |
| **as1** | 17.569 | 19.176 | 20.772 |  | 15.604 | 18.343 | 21.362 | 21.745 |
| **as2** | 19.347 | 19.176 | 19.036 |  | 17.067 | 18.343 | 19.550 | 3.134 |
| **as4** | 18.892 | 19.176 | 19.469 |  | 17.997 | 18.343 | 18.689 | 0.406 |
| **as6** | 18.889 | 19.176 | 19.465 |  | 18.017 | 18.343 | 18.664 | 0.375 |
| **a1_3** | 18.961 | 19.176 | 19.372 |  | 18.078 | 18.343 | 18.588 | 0.215 |
| **a1_5** | 18.985 | 19.176 | 19.354 |  | 17.979 | 18.343 | 18.677 | 0.312 |
| **a2_0** | 19.131 | 19.176 | 19.221 |  | 18.766 | 18.343 | 17.950 | 0.338 |
| **a2_7** | 19.347 | 19.176 | 19.026 |  | 18.612 | 18.343 | 18.105 | 0.181 |
| **a2_14** | 19.068 | 19.176 | 19.282 |  | 17.556 | 18.343 | 19.028 | 1.112 |
| **a3_1** | 19.190 | 19.176 | 19.162 |  | 18.361 | 18.343 | 18.324 | 0.001 |
| **a3_8** | 18.588 | 19.176 | 19.725 |  | 17.336 | 18.343 | 19.302 | 2.581 |
| **a4_9** | 19.398 | 19.176 | 18.977 |  | 18.611 | 18.343 | 18.104 | 0.218 |
| **a5_1** | 19.185 | 19.176 | 19.168 |  | 18.360 | 18.343 | 18.325 | 0.001 |
| **a5_11** | 18.821 | 19.176 | 19.482 |  | 17.615 | 18.343 | 18.972 | 1.144 |
| **a7_2** | 19.032 | 19.176 | 19.313 |  | 18.127 | 18.343 | 18.547 | 0.128 |
| **a8_9** | 19.193 | 19.176 | 19.160 |  | 18.386 | 18.343 | 18.300 | 0.004 |
| **a8_15** | 19.168 | 19.176 | 19.184 |  | 18.318 | 18.343 | 18.364 | 0.001 |
| **a9_8** | 19.139 | 19.176 | 19.208 |  | 18.246 | 18.343 | 18.425 | 0.019 |
| **a10_11** | 19.182 | 19.176 | 19.170 |  | 18.357 | 18.343 | 18.329 | 0.000 |
| **a10_10p** | 19.212 | 19.176 | 19.141 |  | 18.438 | 18.343 | 18.248 | 0.021 |
| **a11_10** | 19.015 | 19.176 | 19.312 |  | 17.925 | 18.343 | 18.695 | 0.343 |
| **a11_16** | 19.170 | 19.176 | 19.182 |  | 18.329 | 18.343 | 18.356 | 0.000 |
| **a12_13** | 19.152 | 19.176 | 19.199 |  | 18.326 | 18.343 | 18.358 | 0.002 |
| **a13_12** | 19.179 | 19.176 | 19.173 |  | 18.345 | 18.343 | 18.340 | 0.000 |
| **a13_17** | 19.176 | 19.176 | 19.177 |  | 18.342 | 18.343 | 18.343 | 0.000 |
| **a14_18** | 19.179 | 19.176 | 19.173 |  | 18.345 | 18.343 | 18.340 | 0.000 |
| **g1** | 13.076 | 19.176 | 30.465 |  | 3.935 | 18.343 | 80.869 | 4281.806 |
| **g2** | 19.404 | 19.176 | 18.964 |  | 18.607 | 18.343 | 18.094 | 0.228 |
| **g3** | 19.028 | 19.176 | 19.382 |  | 20.144 | 18.343 | 16.222 | 7.806 |
| **g4** | 19.839 | 19.176 | 18.645 |  | 18.998 | 18.343 | 17.790 | 1.456 |

Subsequently, we performed two further rounds of MC simulations. In the first, we assessed compensation between the biomass activation parameter g1 of metal uptake and the two rate constants associated with metal uptake: as1 and as2. Specifically, all parameters were kept at their baseline values, while g1 was allowed to vary between 2 and 4, as1 between 0.001 and 0.005 and as2 between 0.001 and 0.2, which corresponded roughly to 50% changes up or down. As the metric of quality, we computed the sum of squared errors (SSE) between the data (Fe and Zn in biomass at Days 10 and 13) and the model. For the baseline parameter values, the residual error is SSE = 0.163. Among 1,000 randomized simulations, only 14 produced a residual error SSE <10; in these simulations g1 was always between 2.85 and 3.54, with as1 and as2 compensating to some degree. Only one simulation resulted in SSE<1. In this case, g1 = 3.363384, as1 = 0.001756859, and as2 = 0.004569567, yielding SSE = 0.755. The highest SSE (=2,640,238) was obtained for g1 = 3.99646, as1 = 0.003835414, and as2 = 0.01971587. The results indicate that g1 must be within a narrower range for low SSE values.

Thus, we repeated this analysis with g1 between 2.8 and 3.5. This time, among 1,000 randomized simulations, 29 led to SSE <10 and two led to SSE<1. Again, some combinations led to very high SSE values. The two simulations with SSE<1 had the parameter combinations

g1 = 2.911408, as1 = 0.00465453, as2 = 0.01252687; SSE = 0.158840611

g1 = 2.891647, as1 = 0.004817988, as2 = 0.01258022; SSE = 0.42538626

They have slightly lower values for g1 and somewhat higher values for as1 and as2 at baseline. Thus, a modest degree of compensation is possible. The extreme sensitivity of the model with respect to these parameters becomes further evident if the values of the first parameter set are simply rounded to two significant digits, which leads to

g1 = 2.9, as1 = 0.0047, as2 = 0.013; SSE = 0.048,

which has an SSE that is less than 1/3 of the SSE without rounding. The relationship between g1 and as1 and as2 is discussed further in a later section and Figure A2.

A further round of massive MC simulations was implemented to determine two additional aspects associated with the baseline parameter set. First, the sensitivity analysis with results in Table A1 focuses on one parameter at a time, thereby excluding compensatory and synergistic effects among metal uptake parameters as they appeared for g1, as1 and as2. Second, given the size of the model we considered it beneficial to outline an ***ensemble*** of models with different parameter combinations but reasonable fits to the pertinent data. Here, the data were again the observed amounts of Fe and Zn in biomass on Days 10 and 13.

We initially permitted all parameter values to vary between 50% and 150% of the baseline values. It turned out again that reasonably good solutions, with SSE<10, required g1 to be between about 2.8 and 3.5, without a single exception. In fact, among 1,000 simulations the lowest SSE with g1 outside this range was 35.22 (g1 = 3.67). Furthermore, no parameter set with g1< 2.69 or g1> 3.68 had an SSE <100 and no parameter set with g1< 2.93 or g1> 3.25 had an SSE <10. Finally, the maximal SSE exceeded 5×10^11^ if g1 was allowed to vary between 50% and 150% of its baseline value.

Based on these preliminary results, we restricted the range of g1 again to [2.8, 3.5]. Among 12,000 simulations with this restriction, 33 simulations had SSE<1 and 141 had SSE<4. This cutoff of SSE = 4 was chosen because it falls well into the range given by the standard deviations in the data for the four datapoints (Fe and Zn in biomass at Days 10 and 13), which is about 2. Indeed, a solution with SSE = 4.03 matches the data rather well (Table A2).

**Table A2: A variation in model parameter values, leading to sum of squared errors,**

**SSE = 4.03, matches the data well**

| **Day** | **Fe Biomass** | |  | **Zn Biomass** | |
| --- | --- | --- | --- | --- | --- |
|  | Observation  Mean + Stand. Dev. | Model |  | Observation  Mean + Stand. Dev. | Model |
| **7** | 4.93 + 1.17 | 4.9 |  | 1.91 + 1.02 | 1.9 |
| **10** | 9.79 + 1.35 | 9.995353 |  | 2.89 + 0.82 | 3.521682 |
| **13** | 19.13 + 2.68 | 20.09183 |  | 18.65 + 3.17 | 17.0662 |

Some (sensitive) parameters have relatively narrow ranges (including g1), whereas other (insensitive) parameters vary throughout the assigned range of +50% of the baseline. These values define the domain of a model ensemble that fits the data well. In fact, every row in a corresponding Excel spreadsheet is a member of this ensemble, if the corresponding SSE falls below a predefined value. Three examples are shown in Table A3, two with very low (0.35, and 0.57) and one with reasonably good SSE (3.0).

**Table A3:**

**Members of a Model Ensemble, Obtained with Monte-Carlo Simulation and**

**Selected by sum of squared errors (SSE)**

| **Parameter** | **g1** | **g2** | **g3** | **g4** | **as1** | **as2** | **as4** |
| --- | --- | --- | --- | --- | --- | --- | --- |
| **Simulation 1** | 2.824503 | -0.3382 | 1.378368 | -0.24488 | 0.00475 | 0.014247 | 16.70861 |
| **Simulation 2** | 3.103867 | -0.23664 | 2.228858 | -0.42972 | 0.002507 | 0.01097 | 16.67486 |
| **Simulation 3** | 2.829182 | -0.17827 | 1.26191 | -0.41408 | 0.00545 | 0.008537 | 17.99509 |

| **as6** | **a1_3** | **a1_5** | **a2_0** | **a2_7** | **a2_14** | **a3_1** | **a3_8** |
| --- | --- | --- | --- | --- | --- | --- | --- |
| 24.16974 | 2.066729 | 2.939573 | 0.05428 | 3.382733 | 0.538492 | 0.125726 | 1.445311 |
| 25.82873 | 2.84669 | 2.511155 | 0.047027 | 3.892636 | 0.267716 | 0.083415 | 1.25689 |
| 27.49748 | 2.344234 | 2.205054 | 0.082101 | 2.320566 | 0.727218 | 0.138867 | 0.805796 |

| **a4_9** | **a5_1** | **a5_11** | **a7_2** | **a8_9** | **a8_15** | **a9_8** | **a10_11** |
| --- | --- | --- | --- | --- | --- | --- | --- |
| 8.154698 | 0.06485 | 2.147374 | 0.610172 | 0.873882 | 1.484649 | 2.905095 | 0.10793 |
| 5.755943 | 0.128524 | 2.274026 | 1.377834 | 0.682348 | 1.323389 | 2.827197 | 0.116286 |
| 7.717063 | 0.132162 | 2.254189 | 1.281121 | 1.203177 | 0.506562 | 2.530251 | 0.058893 |

| **a10_10p** | **a11_10** | **a11_16** | **a12_13** | **a13_12** | **a13_17** | **a14_18** | **SSE** |
| --- | --- | --- | --- | --- | --- | --- | --- |
| 0.091182 | 2.529923 | 0.099907 | 0.086894 | 0.061155 | 0.078242 | 0.119363 | **0.353852** |
| 0.085069 | 2.796289 | 0.110991 | 0.105623 | 0.145682 | 0.097887 | 0.053748 | **0.566974** |
| 0.081452 | 4.555864 | 0.103716 | 0.076527 | 0.056931 | 0.142285 | 0.072062 | **2.995337** |

Inspection of Table A3 demonstrates again that the values of some parameters are not particularly critical. For instance, even among these three instantiations, a8_15 varies throughout its permitted range [0.5, 1.5], whereas others are tight although their permitted range is wider; an example is a11_16, whose permitted range is [0.5, 1.5]. When exploring the ensemble, one should be aware that it is not legitimate to compose parameter sets by averaging each parameter, because this strategy would ignore relationships among subsets of parameters (Ch. 5 in Voit, 2017). An example of such a relationship is shown in Figure A2.


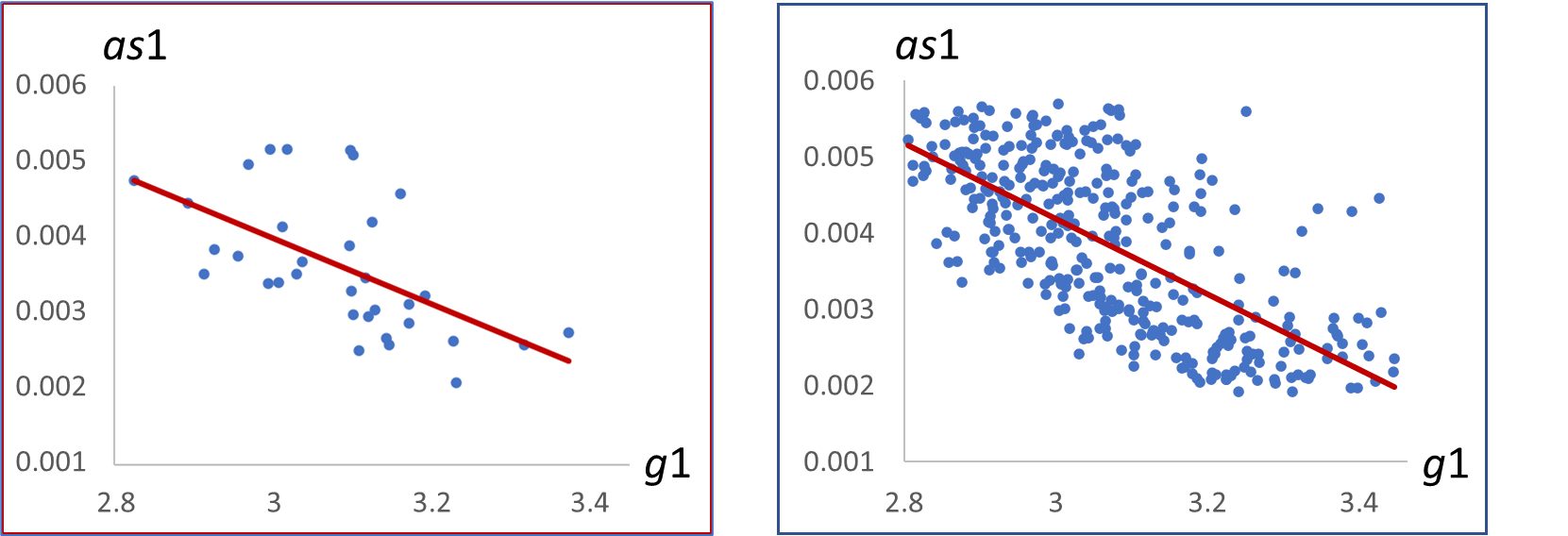


**Figure A2:** Relationship between g1 and as1 for SSE<1 (left) and SSE<10 (right).

One should mention that the notion of an ensemble is not unique but that its “size,” measured in terms of the ranges of the various parameters, depends on the SSE cutoff and is larger for more relaxed SSEs.

In summary, the parameterized model appears to be fairly robust as long as some sensitive parameter values are retained in relatively narrow ranges.

**References**

Alvarez-Vasquez, F., Sims, K. J., Hannun, Y. A., & Voit, E. O. (2004). Integration of kinetic information on yeast sphingolipid metabolism in dynamical pathway models. *Journal of Theoretical Biology*, *226*(3), 265–291. <https://doi.org/10.1016/j.jtbi.2003.08.010>

Curto, R., Sorribas, A., & Cascante, M. (1995). Comparative characterization of the fermentation pathway of Saccharomyces cerevisiae using biochemical systems theory and metabolic control analysis: Model definition and nomenclature. *Mathematical Biosciences*, *130*(1), 25–50. <https://doi.org/10.1016/0025-5564(94)00092-e>

Curto, R., Voit, E. O., Sorribas, A., & Cascante, M. (1998). Mathematical models of purine metabolism in man. *Mathematical Biosciences*, *151*(1), 1–49. <https://doi.org/10.1016/s0025-5564(98)10001-9>

Edelstein-Keshet L. (1988) Mathematical Models in Biology. McGraw-Hill, 1988 (reprinted by SIAM, 2005).

Voit, E.O. (1991). *Canonical Nonlinear Modeling. S-System Approach to Understanding Complexity*. Van Nostrand Reinhold, NY: 1991; p xi+365 pp.

Voit, E. O. (2017). A First Course in Systems Biology. Second Edition, Garland Science, New York, NY, 2017.

Voit, E. O. (2013). Biochemical Systems Theory: A Review. *ISRN Biomathematics*, *2013*, e897658. <https://doi.org/10.1155/2013/897658>

Voit, E. O. (2000). *Computational analysis of biochemical systems: A practical guide for biochemists and molecular biologists*. Cambridge University Press.

Torres, N. V., & Voit, E. O. (2002). *Pathway Analysis and Optimization in Metabolic Engineering*. Cambridge University Press.

Savageau, M. A. (1976). Biochemical systems analysis. A study of function and design in molecular biology. In *ADDISON WESLEY PUBL.* <http://www.scopus.com/inward/record.url?scp=16744368260&partnerID=8YFLogxK>

Savageau, M. A., & Voit, E. O. (1987). Recasting nonlinear differential equations as S-systems: A canonical nonlinear form. *Mathematical Biosciences*, *87*(1), 83–115. <https://doi.org/10.1016/0025-5564(87)90035-6>
